# Long-read whole genome sequencing and comparative analysis of six strains of the human pathogen *Orientia tsutsugamushi*

**DOI:** 10.1371/journal.pntd.0006566

**Published:** 2018-06-06

**Authors:** Elizabeth M. Batty, Suwittra Chaemchuen, Stuart Blacksell, Allen L. Richards, Daniel Paris, Rory Bowden, Caroline Chan, Ramkumar Lachumanan, Nicholas Day, Peter Donnelly, Swaine Chen, Jeanne Salje

**Affiliations:** 1 Wellcome Centre for Human Genetics, University of Oxford, Oxford, United Kingdom; 2 Mahidol-Oxford Tropical Medicine Research Unit, Faculty of Tropical Medicine, Mahidol University, Bangkok, Thailand; 3 Centre for Tropical Medicine and Global Health, Nuffield Department of Medicine, University of Oxford, Oxford, United Kingdom; 4 US Naval Medicine Research Center, Silver Spring, Maryland, United States of America; 5 Swiss Tropical and Public Health Institute, Basel, Switzerland; 6 Faculty of Medicine, University Basel, Basel, Switzerland; 7 Pacific Biosciences, 1305 O’Brien Drive, Menlo Park, California, United States of America; 8 Department of Statistics, University of Oxford, Oxford, United Kingdom; 9 Department of Medicine, Division of Infectious Diseases, Yong Loo Lin School of Medicine, National University of Singapore, Singapore; 10 Genome Institute of Singapore, A*STAR, Singapore; 11 Public Health Research Institute, Rutgers Biomedical and Health Science, Newark, New Jersey, United States of America; Instituto de Pesquisas Veterinarias Desiderio Finamor, BRAZIL

## Abstract

**Background:**

*Orientia tsutsugamushi* is a clinically important but neglected obligate intracellular bacterial pathogen of the Rickettsiaceae family that causes the potentially life-threatening human disease scrub typhus. In contrast to the genome reduction seen in many obligate intracellular bacteria, early genetic studies of *Orientia* have revealed one of the most repetitive bacterial genomes sequenced to date. The dramatic expansion of mobile elements has hampered efforts to generate complete genome sequences using short read sequencing methodologies, and consequently there have been few studies of the comparative genomics of this neglected species.

**Results:**

We report new high-quality genomes of *O*. *tsutsugamushi*, generated using PacBio single molecule long read sequencing, for six strains: Karp, Kato, Gilliam, TA686, UT76 and UT176. In comparative genomics analyses of these strains together with existing reference genomes from Ikeda and Boryong strains, we identify a relatively small core genome of 657 genes, grouped into core gene islands and separated by repeat regions, and use the core genes to infer the first whole-genome phylogeny of *Orientia*.

**Conclusions:**

Complete assemblies of multiple *Orientia* genomes verify initial suggestions that these are remarkable organisms. They have larger genomes compared with most other Rickettsiaceae, with widespread amplification of repeat elements and massive chromosomal rearrangements between strains. At the gene level, *Orientia* has a relatively small set of universally conserved genes, similar to other obligate intracellular bacteria, and the relative expansion in genome size can be accounted for by gene duplication and repeat amplification. Our study demonstrates the utility of long read sequencing to investigate complex bacterial genomes and characterise genomic variation.

## Author summary

*Orientia tsutsugamushi* is an obligate intracellular bacterial pathogen, and the causative agent of the human disease scrub typhus. This vector-borne bacterial infection can be life-threatening if not treated rapidly with appropriate antibiotics. *Orientia* is endemic across large parts of Asia and is estimated to infect at least one million people per year. *O*. *tsutsugamushi* has a highly unusual genome. At 2.1 Mbp in length, it is almost double the size of most other obligate intracellular bacterial genomes, which have undergone extensive genome reduction in their adaptation to the intracellular lifestyle. The comparatively expanded genome of *Orientia* is accounted for by an abundance of repeated DNA elements. Indeed, the genome of *Orientia* is the most highly repeated bacterial genome reported to date. As a consequence of this, it has been difficult to generate complete genome assemblies of *Orientia* using short-read sequencing technologies. In the current report, the authors use Pacific Biosciences long-read sequencing to generate six complete genomes of diverse strains of *O*. *tsutsugamushi*. By comparing these genomes, the authors show that there is very little colinearity in the ordering of genes within the genome, even between closely related strains. This lack of synteny suggests that there have been frequent events of massive chromosomal rearrangement in this species, although the underlying mechanisms for this have not been shown. The genome sequences and analysis reported here will benefit those working on various aspects of the biology of this clinically important but neglected bacterial species.

## Introduction

*O*. *tsutsugamushi* is an obligate intracellular bacterial pathogen of the order Rickettsiales, family Rickettsiaceae which causes the life-threatening human disease scrub typhus. *Orientia* is transmitted by *Leptotrombidium* mites that occasionally feed on humans during the larval stage of development (“chiggers”), inoculate bacteria into the skin, and initiate infection. *Orientia* is maintained in mite populations by transovarial transmission. The mites normally feed only once on a vertebrate host, and cannot transmit bacteria directly from one host to another [1]. Bacteria propagate within endothelial cells, dendritic cells and monocytes at the site of inoculation, sometimes resulting in a visible red skin feature called an eschar [2]. Bacteria subsequently spread through the endothelial and lymphatic systems to cause a systemic infection characterised by lymphadenopathy, headache, fever, rash and myalgia, which typically begin 7–10 days after inoculation. The non-specificity of these symptoms makes scrub typhus difficult to diagnose based purely on clinical observations, and this is an important reason why the prevalence of scrub typhus has been historically under-recognised. Scrub typhus has now been shown to be a leading cause of severe fever and sepsis in studies in Thailand, India, China, Laos and Myanmar [3] and untreated or severe cases are associated with CNS infection, morbidity and death [4,5]. Locally acquired cases of scrub typhus have been reported in South America and the Middle East [6,7], suggesting that the global burden of this disease may stretch beyond the traditionally known endemic areas of Asia and Northern Australia [3].

*O*. *tsutsugamushi* is distinct from other members of the Rickettsiaceae. The genus *Orientia* currently includes two known species, *O*. *tsutsugamushi* and *O*. *chuto*, the latter represented to date by a single strain inoculated from a patient with a febrile illness contracted in Dubai [6]. High antigenic diversity among strains of *O*. *tsutsugamushi* is reflected in the poor immunological protection that recovered patients exhibit towards strains different from their original infection and this has hampered efforts towards vaccine development.

Despite its importance as a pathogen, few genomic analyses of *O*. *tsutsugamushi* have been published. The first whole genome sequence, Boryong, [8] reported a proliferation of type IV secretion systems in a repeat-dense genome of which 37.1% comprised identical repeats. A comparison of Boryong and the second complete genome, Ikeda [9], revealed similar repeats present in each genome, dominated by an integrative and conjugative element named the *O*. *tsutsugamushi* amplified genetic element (OtAGE), and identified a core genome of 520 genes shared between the two *O*. *tsutsugamushi* strains and the 5 available sequences of other *Rickettsia* [9]. Extensive genomic reshuffling was thought to have been mediated by amplification of repetitive sequences.

In comparison to other *Rickettsiae*, many of which have small and extremely stable genomes, *O*. *tsutsugamushi* has a large genome with an extraordinary proliferation of repeat sequences and conjugative elements. Some of the conjugative elements present in multiple copies across the genome are homologues of a gene cluster found in a single copy in *Rickettsia bellii*. Many of the genes in these elements are fragmented, suggesting they are non-functional [10]. Other intracellular pathogens also contain repetitive elements, such as the mobile genetic elements in *Wolbachia* [11] and the tandem intergenic repeats in *Ehrlichia ruminantum* [12]. These mechanisms may evolve to increase genetic variability and aid immune evasion in bacteria which cannot easily take up novel DNA.

Larger collections of *O*. *tsutsugamushi* strains have been extensively studied using MLST and sequence typing of the *groES* and *groEL* [13] genes, and the outer membrane proteins 47kDa (also called HtrA or TSA47) [14] and 56kDa (also called OmpA or TSA56) [15] genes. The 56kDa and 47kDa genes are highly immunogenic in human patients and animal models and have long been investigated as candidates for vaccine design, but high levels of diversity between strains, especially in the 56kDa gene, have limited the potential of developing a universal vaccine based on these epitopes.

Multiple studies in South East Asia have looked at the diversity of strains by MLST and 56kDa typing, and shown a high level of diversity, with many MLSTs unique to an individual strain [16–19]. Work in Thailand and Laos has shown recombination between MLSTs, as well as evidence for multiple infections in individual patients, implying that multiple strains may co-exist in mites [16]. Comparisons of *56kDa* typing with MLST [16] and *47kDa* [14] also show low congruence between methods, suggesting that single gene typing of *Orientia* may not represent the true relationships between strains; by extension, a 7-gene MLST scheme may not capture the full set of genomic relationships among strains.

Attempts to generate complete *O*. *tsutsugamushi* genomes by whole genome sequencing have been limited by the difficulties of sequencing and assembling a repeat-dense genome, and no further genomes have been completed since the Boryong and Ikeda genomes in 2008. Current draft assemblies are fragmented with over 50 contigs per genome, and vary in size–the two assemblies of the genome of *O*. *tsutsugamushi* str. Karp available on Genbank are 1,459,958bp (https://www.ebi.ac.uk/ena/data/view/LANM01000000) and 2,022,909bp (https://www.ncbi.nlm.nih.gov/nuccore/LYMA00000000)[20] in length, suggesting that assemblies are either incomplete, or have problems caused by the misassembly of repeats or the inclusion of contaminating sequences.

In this work, we have used Pacific Biosciences long-read sequencing to assemble six complete genomes of *O*. *tsutsgamushi* strains representing a range of geographical origins and serotypes. This expanded genomic perspective will contribute to our understanding of the phylogeography and epidemiology of this species, as well as contribute to more detailed studies of virulence mechanisms.

## Methods

### Bacterial propagation

All experiments were performed using *O*. *tsutsugamushi* grown in the mouse fibroblast cell line L929 (European Collection of Authenticated Cell Cultures 85103115). Uninfected L929 cells were grown in 25 cm^2^ and 75 cm^2^ plastic flasks at 37 ^o^C and 5% CO_2_, using DMEM or RPMI 1640 (Thermo Fisher Scientific, USA) media supplemented with 10% FBS (Sigma) as described previously (Giengkam et al 2015). Infected L929 cells were grown in the same way, but at 35 ^o^C. Frozen stocks of bacteria were grown for 5 days, then the bacterial content was calculated using qPCR against the bacterial gene TSA47 [21]. Bacteria were isolated onto fresh L929 cells in 75 cm^2^ flasks at an Multiplicity of Infection of 10:1 and then grown for an additional 7 days. At this point bacteria were isolated from host cells and prepared for DNA extraction.

### DNA extraction

The supernatant were removed from infected flasks and replaced with 6–8 ml pre-warmed media. Infected cells were harvested by mechanical scraping and then lysed using a bullet blender (BBX24B, Bullet Blender Blue, Nextadvance, USA) operated at power 8 for 1 min. Host cell debris was removed by centrifugation at 300xg for 3 minutes, and the supernatant was filtered through a 2.0 μm filter unit (Puradisc, GE Whatman, USA). 10 μl of 1.4 μg/μl DNase (Deoxyribonuclease I from bovine pancreas, Merck, UK) was added per 1 ml of bacterial solution, then incubated at room temperature for 30 minutes. This procedure removed excess host cell DNA. The bacterial sample was then isolated by centrifugation at 14,000xg for 10 min at 4 ^o^C, and washed two times with 0.3M sucrose (Merck). After the washing steps were completed DNA was extracted using a QIAGEN DNeasy Blood & Tissue Kit (QIAGEN, UK) following the manufacturer’s instructions.

Purified DNA samples were analysed by gel electrophoresis using 0.8% agarose gel, in order to assess the DNA integrity. The yield of genomic DNA was quantified using a nanodrop (Nanodrop 2000, Thermo Scientific, UK) and Qubit Fluorometric Quantitation (Qubit 3.0 Fluorometer, Thermo Scientific).

### Sequencing

SMRTBell templates were prepared from purified *Orientia* genomic DNA according to PacBio’s recommended protocols (PacBio, USA). Briefly, 20kb libraries were targeted; enrichment for large fragments was done using BluePippin (Sage Science, USA) size selection method or successive Ampure (Beckman Coulter, USA) clean-ups, depending on the original DNA size distribution and quantity, as recommended by PacBio. SMRTBell templates were sequenced on a Pacific Biosciences RSII Sequencer using P6 chemistry with a 240min run time. Genomes were assembled using the RS_HGAP_Assembly.3 protocol from the PacBio SMRTPortal (version 2.3.0), with initial polishing performed on trimmed initial assemblies using the same raw sequencing data with the RS_Resequencing.1 protocol. Each assembly was further polished using paired-end reads sequenced on an Illumina Miseq machine. Sequencing information and data availability for each sample can be found in S1 Supporting Information. For each assembly, the corresponding Illumina reads were aligned to the PacBio assembly using Stampy v1.0.23 [22]. Pilon v1.16 [23] was then used to generate a final genome, and corrected 2 to 265 errors in the assemblies, with the majority of the errors being single base deletions at the end of A or T homopolymer runs. All genomes were rotated and reverse complemented as needed so that the predicted start codon for the dnaA gene formed the first nucleotide in the genome sequence. Sequencing and assembly statistics can be found in Table 1 and S1 Supporting Information. The Boryong, Ikeda, and non-*Orientia* Rickettsial genomes used in this study were obtained from NCBI (S1 Supporting Information).

**Table 1:** Overview of *O*. *tsutsugamushi* strains.

Strain	Serotype	Original Source	Source in this study	Reference	Genome length (bp)	Contigs	GC percentage	Errors corrected by Illumina sequencing
Boryong*	Boryong	South Korea, human patient, 1980s	-	[50]	2,127,051	1	31	-
Ikeda*	Ikeda	Japan, human patient, 1979	-	[51]	2,008,987	1	31	-
Karp	Karp	New Guinea, human patient, 1943	Naval Medical Research Centre (NMRC)	[52]	2,469,803	1	31	48
Kato	Kato	Niigata, Japan, human patient, 1955	NMRC	[52]	2,319,449	1	31	5
Gilliam	Gilliam	Indian-Burmese border, human patient, 1943	NMRC	[53]	2,465,012	1	31	7
TA686	TA686	Thailand, animal (*Tupaia glis)*, 1963	NMRC	[52]	2,254,485	1	31	28
TA763	TA763	Thailand, animal *Rattus rajah)*, 1963	NMRC	[52]	2,089,396	8	31	88
FPW1038	TA716	Thailand-Burmese border, human patient (pregnant), 2010	Mahidol-Oxford Research Centre (MORU)	[54]	2,035,338	25	31	265
UT76	Karp	Udon Thani, Thailand, human patient, 2003	MORU	[55]	2,078,193	1	30	2
UT176	Karp	Udon Thani, Thailand, human patient, 2004	MORU	[56]	1,932,116	1	30	13

Bacterial strains used in this study and assembly statistics for the 10 assemblies used in this analysis. Genomes marked with * are previously-assembled reference strains.

The finished assemblies were annotated using Prokka v1.11 [24], using a custom database created from the Boryong and Ikeda strains, which were previously annotated using the NCBI prokaryotic annotation pathway. The Boryong and Ikeda strains were re-annotated using Prokka for consistency with the other samples. Short gene names for all non-hypothetical gene products were checked manually (607 products). Where genes names were present for Boryong and/or Ikeda a consensus name based on these was selected. Where no short name was available, the long gene name was searched for in *E*. *coli* using the UniProt database, and where a single and unambiguous match was selected this was used. In cases of ambiguity the protein sequence from *Orientia* was used in a BLAST search against *E*. *coli*, *R*. *rickettsii* and *H*. *sapiens* and the short name of the closest match was selected. The key *Orientia* genes *TSA56*, *TSA47*, *TSA22*, *ScaA*, *ScaC*, *ScaD*, and *ScaE* were also manually annotated by taking known protein sequences from the UT76 strain and using BLAST to find the homologous genes in the other strains and give them the correct names.

Repetitive regions of the genome were defined as regions of at least 1000bp in length which had a match with another 1000bp region with up to 100 differences (mismatches, insertions, and deletions) allowed. The repetitive regions were identified with Vmatch [25].

The core and accessory genome was identified using Roary [26] with a threshold of 80% sequence identity required to consider two sequences part of the same gene group. Core genes were defined as genes present in every sample and as a single copy in every sample. The accessory genes identified using Roary were re-clustered using CD-Hit [27,28] with a cutoff of 80% identity across 95% of the length of the shortest protein to identify accessory genes which were truncated copies of other proteins. The correlation between gene order in each pair of samples was calculated by taking the order of the genes relative to the Karp strain and calculating the Spearman’s rank coefficient between each pair. COG categories were assigned using RPS-BLAST to find matches in the NCBI Conserved Domain Database [29] and assigning a COG category to these using cdd2cog [30]. Repeat genes were identified using protein clusters generated by CD-Hit to find gene groups which were present at more than 1 copy. CD-Hit was used to identify protein clusters on the proteins predicted by Prokka with a cutoff of 80% identity across 90% of the length of the shortest protein. Pseudogenes were identified from CD-Hit protein clusters where at least one protein was a truncated version of the longest protein in the group. As pseudogenes which are truncated at the 5’ end will not be annotated by Prokka, tBLASTn [31] was using to screen for any additional pseudogenes in non-genic regions by searching for BLAST hits with protein identity > = 80% and an E-value <10^−15^. This method found a further 26–37 pseudogenes per strain.

**Fig 1:**
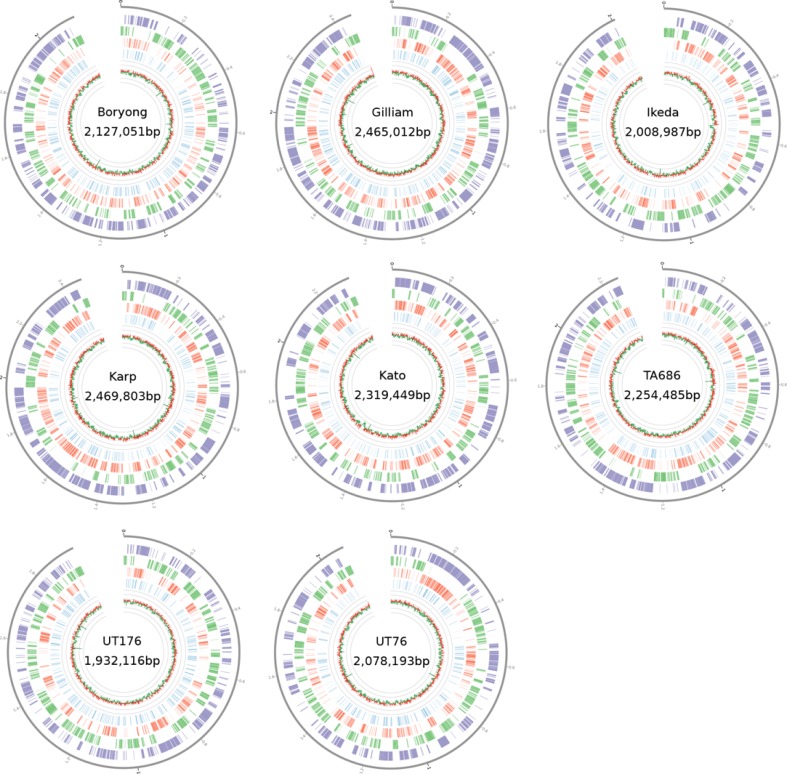
Ring diagrams for all single-contig strains. From outermost feature in each genome, moving inwards: repetitive regions are shown in purple, core genes in green, repeat genes in red and pseudogenes in blue. The track shows the GC percentage in windows of 1000bp. Values above the median GC are in green, and values below the median GC are in red.

Further analysis used BioPython [32] and the GenomeDiagram package [33]. Fig 1 was created with Circos [34]. Statistical tests were carried out in R [35] and the Python SciPy library [36].

Phylogenies were inferred using Maximum Likelihood methods in RaxML [37] under the GAMMA model of rate heterogeneity and bootstrap values calculated using the rapid bootstrap method. The input sequences were aligned with Clustal Omega [38] (for the 56kDa/46kDa/MLST trees) or using the MAFFT alignments produced by Roary (for the core gene tree). Phylogenetic trees were drawn using the ape [39] and phytools [40] R packages, and Robinson-Foulds distances were calculated using the phangorn [41] R package.

## Results

### Sequencing, assembly, and annotation

Eight genomes (Table 1) were assembled using the PacBio reads to perform initial genome assembly and Illumina sequencing reads to polish the genomes and reduce errors. Six of the eight genomes could be assembled into a single finished contig, while two genomes remain in multiple contigs (Table 1). In addition, two previously assembled references genomes, *O*. *tsutsugamushi* str. Boryong and *O*. *tsutsugamushi* str. Ikeda, were incorporated into our analysis. The genome size ranges from 1.93Mb to 2.47Mb, and the GC content for all strains is consistent at 30–31%. We assessed the genomes to identify core genes shared between all genomes, and look for repetitive regions and repeat genes in each strain. Fig 1 plots the genetic elements of each complete genome.

The number of predicted genes in each strain ranges from 2086 (UT176) to 2709 (Gilliam) (Table 2) and is highly correlated with genome size (Spearman’s correlation coefficient 0.94, p < 2.2x10^-16^). A function could not be assigned, by similarity to reported sequences, to 325–547 genes (16 to 22% of the identified coding regions) in each strain.

**Table 2:** Predicted gene numbers in different strains of *O*. *tsutsugamushi*.

Strain	Genes	Annotated as hypothetical
Boryong	2443	547
Ikeda	2186	417
FPW1038	2198	369
Gilliam	2709	463
Karp	2578	470
Kato	2406	465
TA686	2546	474
TA763	2212	396
UT76	2247	420
UT176	2086	325

Number of genes predicted in each strain after annotation with Prokka, and the number of genes which were annotated as hypothetical. The Boryong and Ikeda strains were reannotated with Prokka for consistency between strains.

### Core genome analysis

**Fig 2:**
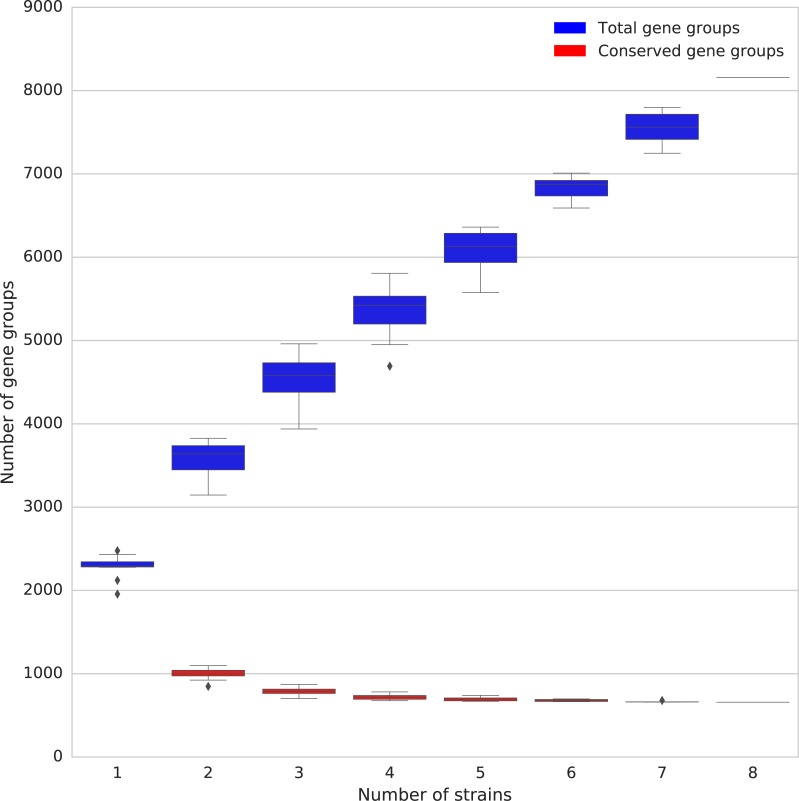
The number of core gene groups and the total number of gene groups (including the core gene groups) as more strains are added to the analysis. Boxplots represent all possible combinations of the number of strains given on the x-axis.

The set of 8 complete, single-contig genomes was used to identify core genes (present in all genomes) and accessory genes (present in a subset of genomes), using the criterion that all members of a group of putative orthologues should be at least 80% identical to all other members of the group. The number of predicted genes and total genome size for the unfinished genomes was comparable with those in the finished genomes, which suggests that while the assemblies are not assembled into a single contig they are not missing large pieces of the genome sequence of these strains. However, since we are unable to verify that they contain the complete genome, we have excluded them from the core gene analysis to avoid concluding that genes are not in the core genome based on their absence from these assemblies. A total of 657 genes were present in all 8 strains and therefore form a putative core genome, while 2812 gene clusters (which may contain multiple genes from a single strain) were present in 2–7 of the 8 strains, and a further 4687 gene clusters were found in a single strain. The 657 core genes make up 28–35% of the genome of each strain (S1 Supporting Information). The number of core genes does not continue to decrease as more genomes are added to the analysis, suggesting that the core genome of *Orientia* can be defined with 8 representative genomes (Fig 2). In the initial analysis with Roary, the total number of gene clusters continued to grow, suggesting an open pan-genome. However, while the total number of distinct gene clusters continues to grow, many of these clusters contain genes which are annotated with the same function as previously observed gene clusters. Of the 6050 clusters where a function can be assigned to one or more gene, there are only 122 different predicted gene products, many of them conjugal transfer proteins, transposases, DNA helicases, and other functions shared by genes known to be part of the *O*. *tsutsugamushi* amplified genetic element identified in the Ikeda strain [42]. Re-clustering these accessory genes using a more lenient length cut-off to determine clusters allows genes which are only a match to part of a gene sequence to cluster together to include more truncated and fragmented copies of genes. A comparison between the standard Roary clusters and this lenient clustering shows that the number of accessory gene clusters continues to increase, but at a slower rate (S1 Supporting Information). The number of gene products remains constant at 122 no matter how many strains are included in the analysis. This suggests that the increase in non-core gene clusters is mainly due to further duplication and truncation of existing genes, rather than by the import of novel genes.

### Genome synteny

**Fig 3:**
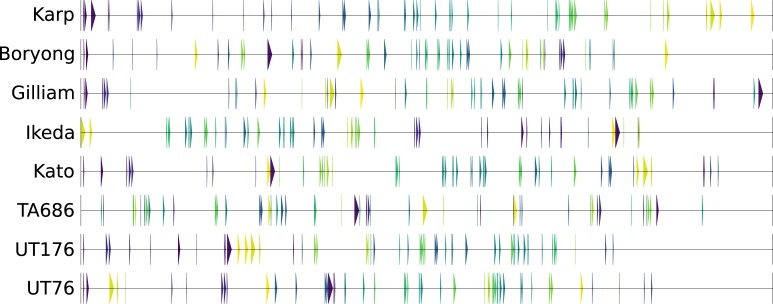
Each arrow represents the location of a core gene island containing one or more core genes which are conserved in the same order within an island across all strains. The arrows are coloured relative to their order in the Karp genome.

With the completed genomes produced by long read sequencing, the synteny of the genomes can be investigated. Previous work on the Boryong and Ikeda genomes showed extensive genome shuffling between the two strains. Analysis of the order and grouping of the core genes which are conserved in each genome shows that the genome has undergone massive rearrangement, with the core genes found in ‘core gene islands’ with repeat regions interspersed between these islands. The 657 core genes are present in 145–157 distinct islands, of which only 51 are conserved (defined as the same genes present in the same order, including single member islands) in all genomes. Fig 3 shows the position and ordering of these conserved core gene islands which are maintained in all samples relative to the position and ordering in the Karp strain. The correlation between gene order in each pair of samples is shown in S1 Supporting Information. A value close to 0 shows low correlation in gene order, while values closer to 1 show higher correlation in gene order. As there are differences in the correlation of gene order between strains, this suggests that the process of genome rearrangement is happening in multiple steps and not as a single event.

The identities of genes present on conserved islands is shown in S1 Supporting Information. Conserved islands range from 1–13 genes in size, with larger islands often containing genes linked by plausible biological functions. For example, groups 3 and 6 include a number of cell division and peptidoglycan biosynthesis genes (including *mraY*, *murF*, *murE*, *pbp*, *ftsL*, *dnaJ* and *dnaK* in group 3 and *murC*, *murB*, *ddl* and *ftsQ* in group 6) and groups 31 and 32 include a number of 30S and 50S ribosomal proteins. Analysis of the number of conserved islands shared between samples shows that the number of conserved islands continues to decrease as more genomes are included (Figure S3 in S1 Supporting Information), and suggests that gene order and clustering is not always constrained in *O*. *tsutsugamushi*. There is no difference seen in the size of the islands between conserved and non-conserved islands (Figure S4 in S1 Supporting Information) (two-sample Kolmogorov-Smirnov test D = 0.085, p-value = 0.86), the nucleotide diversity between genes in the two categories of islands (two-sample Kolmogorov-Smirnov test D = 0.052, p-value = 0.86) (Figure S5 in S1 Supporting Information), or the Clusters of Orthologous Groups (COG) categories assigned to genes in the two island categories (Chi-squared test χ^2^ = 15.03, p = 0.82) (Figure S6 in S1 Supporting Information).

### Repeats and pseudogenes

The genomes of *O*. *tsutsugamushi* are known to be highly repetitive, including a highly amplified genetic element known as the *O*. *tsutsugamushi* amplified genetic element (OtAGE), as well as other transposable elements.

Our results emphasise the large number of repeated genes and regions, including many genes related to the Type IV secretion system. The total proportion of the genome which is repetitive (see Methods for our definition of repetitive) differs markedly from 33% in UT176 to 51% in Gilliam (S1 Supporting Information). In contrast, the extremely compact (and therefore non-repetitive) *Rickettsia typhi* genome is 0% repetitive by our measure and even, intriguingly, the *Rickettsia* endosymbiont of *Ixodes scapularis*, known to encode multiple copies of the same repetitive element found in *Orientia* [43], is 20% repetitive in our analysis, despite our methods giving more conservative figures than previously determined for the Ikeda strain [42].

We identified 530 groups of repeat genes containing 12043 genes present in multiple copies in at least one strain. Of the 530 groups, 427 represent genes found in multiple strains, while 103 are found only in a single strain. Despite clustering in 530 groups, the genes have only 66 different functional products, as is expected from the earlier results looking at all the non-core genes. The repeat genes are mainly transposase and conjugal transfer genes, similar to those previously reported in the OtAGE (S1 Supporting Information), and cluster into genetic elements which are interspersed between the core genes. Many of these genes are present in high copy number, with all strains carrying over 200 transposases and 300 conjugal transfer genes and gene fragments. These 530 repeat elements occupy 35–47% of the *O*. *tsutsugamushi* genome and represent 57–67% of the genes in these genomes (S1 Supporting Information).

*O*. *tsutsugamushi* genes are known to exhibit high levels of pseudogenisation and gene decay. We searched for pseudogenes in each genome, and identified up to 484 pseudogenes per strain (S1 Supporting Information). This is lower than previously reported in Ikeda, but due to methodological differences the figures cannot be directly compared. We also assessed whether the pseudogene had been caused by truncation at the 5’ or 3’ end of the sequencing, or by frameshift. We did not see a larger number of pseudogenes caused by frameshifts in the genomes new to this study compared to the Boryong and Ikeda strains, suggesting that we do not have a large number of frameshift errors caused by PacBio sequencing.

### Phylogenetics

**Fig 4:**
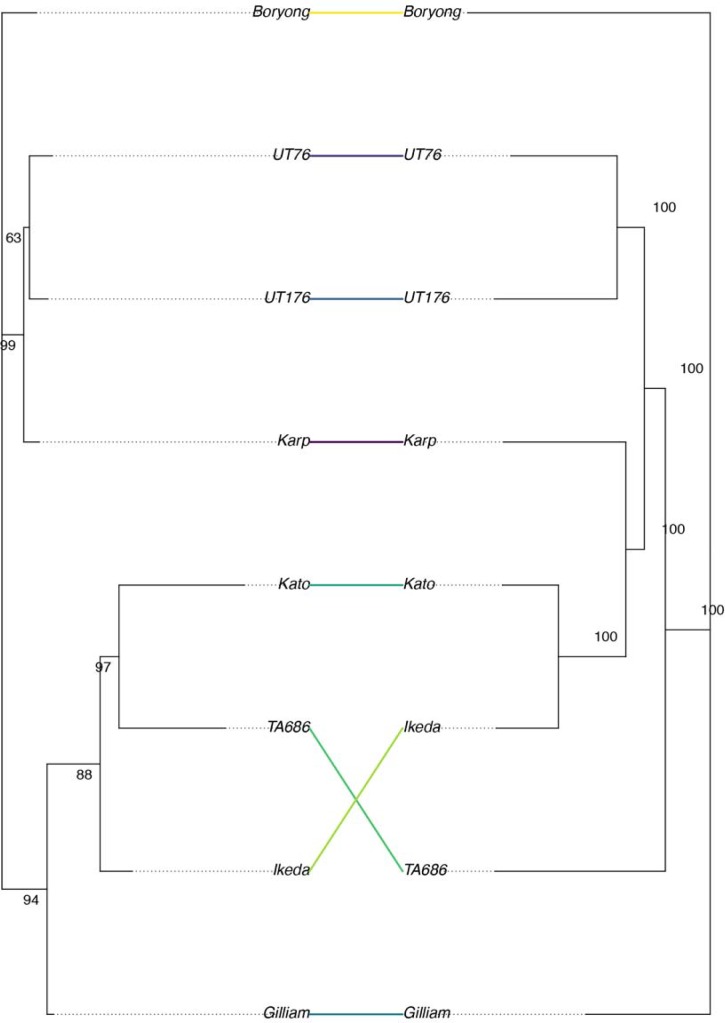
Phylogenetic trees generated from the 56kDa antigen sequence (left) and the sequence of the 657 core genes (right). The tree was inferred using the maximum likelihood method implemented in RaxML, and bootstrap values were calculated with the RaxML rapid bootstrap method.

A phylogenetic tree was constructed using the core genes from each strain. This can be compared to trees built using the 56kDa (Fig 4) and 47kDa (Figure S7 in S1 Supporting Information) genes, which are often used for phylogenetic analysis of *O*. *tsutsugamushi*, or to trees built using the 7 MLST genes from the MLST scheme developed in Sonthayanon et al., 2010 (Figure S8 in S1 Supporting Information). *Orientia* strains are commonly based on their similarity to reference strains, either from phylogenetics or serology. Compared to the 56kDa tree, the core gene tree suggests the Kato and Ikeda strains are more closely related to the Karp, UT176, and UT76 strains than the TA686 and Gilliam strains (Fig 4). Robinson-Foulds distances between trees are shown in S1 Supporting Information; for this small number of strains, the distance is lowest between the 47kDa tree and the core genome tree.

## Discussion

We present the first large-scale genomic comparison of O. tsutsugamushi, a bacterium which is important both for the study of human disease and for its unique insights into genome evolution.

Previous studies of O. tsutsugamushi genomes have used BAC cloning and Sanger sequencing to produce complete genomes [8,42], or have used next-generation sequencing strategies which have produced only incomplete and fragmented genomes [20]. We demonstrate that a combination of PacBio and Illumina sequencing is sufficient to produce a single-contig genome, allowing us to study the gene content and synteny of this organism. For the two genomes which could not be assembled into single contigs in our study (FPW1038 and TA763), we found that the sequencing produced fewer reads at the high end of the length distribution. This suggests that given the highly repetitive nature of the *O*. *tsutsugamushi* genome, the DNA preparation and sequencing methods must be carefully chosen to produce very long reads in order to produce complete assemblies. We used Illumina sequencing to correct errors in our genomes, which was vital to reduce the number of homopolymer errors, which could otherwise suggest frameshift errors and affect gene annotation. While the fewest errors we corrected in a strain was two, this is likely an underestimate as no Illumina reads map with high confidence to 5–15% of the genome due to the repeats, and regions where Illumina reads cannot map will be impossible to correct using this approach. While our analysis shows small differences when quantifying the extent of the repeat regions and repeat gene families in *Orientia* compared to previous work, a direct comparison is difficult due to differences in methodology between analyses.

Owing to the difficulties of producing complete genomes, most previous work has relied on single gene or MLST studies to investigate the genetic diversity of *O*. *tsutsugamushi*. We demonstrate that phylogenies generated from limited data are substantially different from those produced from the whole core genome. The common practice of grouping *Orientia* strains into ‘Karp-like’ or ‘Gilliam-like’ groups based on the genotype of the 56kDa antigen may not give an accurate view of the relatedness of these strains, especially when recombination is taken into account, although this may still be important when considering immune response. The core gene set we have defined can be sequenced using Illumina reads alone and will allow future studies to perform large-scale phylogenetic analysis of *Orientia* without needing long-read sequencing. We have not investigated the effect of recombination in this study due to the difficulties of analyzing recombination in a highly fragmented and rearranged genome, and it is possible that the core gene tree we present does not represent the true phylogeny of these strains.

Previous work has demonstrated limited synteny between the two reference strains of *O*. *tsutsugamushi*, but we extend this to demonstrate that there is minimal synteny between any known *O*. *tsutsugamushi* genome. The pattern of core gene islands separated by transposable elements and repeats suggests a repeat-mediated system of chromosome rearrangement. It is unclear whether this is a gradual process of genome rearrangement, or whether the genome is being broken apart and rearranged rapidly, similar to chromothripsis or the chromosome repair of *Deinoccocus radiodurans* after exposure to ionizing radiation. In *Deinococcus*, it is thought that RecFOR pathway is particularly important for DNA repair, and it has no homologues to RecB or RecC [44]. Similarly, in *Orientia*, the core genome does not contain RecB or RecC, but does contain the RecFOR pathway genes, indicating this alternative DNA repair pathway may also be important. Longitudinal studies of *O*. *tsutsugamushi* genomes during passage or infection may be needed to determine the speed and processes of genome rearrangement in *Orientia*.

We report a core genome of only 657 genes, compared to the 519 previously reported as the core genome shared between *Orientia* and five other sequenced *Rickettsia*, but similar to the 665 core genes shared between the Ikeda and Boryong strains in a previous study [9]. Differences in methodology may lead to the reporting of different core gene sets, but more interesting is the pattern of core genome islands separated by amplified repeat regions, and the lack of conservation in the ordering and clustering of the core genes.

All of the *Orientia* genomes show high repetitiveness, which we measured as both non-unique regions of the genome, and genes which are present in multiple copies (some of which may be truncated). The genomes of intracellular bacteria tend towards genome reduction and gene loss [10,45], but maintain degraded genes and accumulate non-coding DNA. The transition to intracellularity leads to smaller effective population sizes, as the bacteria are sequestered inside cells [46]. As the majority of mutations have a neutral of slightly deleterious effect, they will be removed by purifying selection; however, purifying selection is less effective in smaller bottlenecked populations such as intracellular bacteria, which will lead to an accumulation of slightly deleterious mutations and an increased rate of sequence evolution [47]. The expansion of the OtAGE (and other mobile elements) throughout the *Orientia* lineage appears to be another consequence of the decreased role of selection on *Orientia* in its intracellular niche, again leading to accelerated sequence evolution of the genome through rearrangement and gene loss. This is supported by the finding that the diversity of gene repertoire between strains of *O*. *tsutsugamushi* is partly due to the duplication and truncation of existing genes. The amplification of a transposable element has been seen in Rickettsial [43] and non-Rickettsial [48] species, but it is not known whether this is associated with rearrangement of the genome in other species. High levels of genome plasticity and recombination have also been seen in fungal endobacteria, and are thought to be a defence against Muller’s ratchet [49]

In conclusion, we report the generation of six complete and a further two draft genomes from a diverse set of strains of the important but neglected human pathogen *O*. *tsutsugamushi*. This set includes the major reference strains Karp, Kato and Gilliam, and will serve as a valuable resource for scientists and clinicians studying this pathogen, in particular supporting future work on *Orientia* genomics, vaccine development, and cell biology. The new genomes reported here confirm the status of *Orientia* as one of the most fragmented and highly repeated bacterial genomes known, and exciting questions remain regarding the mechanisms and timeframes driving the evolution of these extraordinary genomes.

## Supporting information

S1 Supporting Information File containing all supporting figures and table. (PDF)
