# Supplementary material for: Long-read whole genome sequencing and comparative analysis of six strains of the human pathogen Orientia tsutsugamushi
Source: PLoS Negl Trop Dis. 2018 Jun 6;12(6):e0006566. doi: 10.1371/journal.pntd.0006566 (PMC6005640; doi:10.1371/journal.pntd.0006566)
Supplement: S1 Supporting Information — (PDF) [file pntd.0006566.s001.pdf]

Supplementary Figures and Tables

Long-read whole genome sequencing and comparative analysis of six strains of the human pathogen *Orientia tsutsugamushi*

Elizabeth M. Batty[a,b,c], Suwittra Chaemchuen[b], Stuart D. Blacksell[b,c], Allan Richards[j], Daniel Paris[b,c,d,e], Rory Bowden[a], Caroline Chan[f], Ramkumar Lachumanan[f], Nicholas Day[b,c], Peter Donnelly[a,g], Swaine L. Chen[h,i], Jeanne Salje[b,c#]

Wellcome Centre for Human Genetics, University of Oxford, Oxford, OX1 7BN, UK[a] ;

Mahidol-Oxford Tropical Medicine Research Unit, Faculty of Tropical Medicine, Mahidol University, Bangkok, Thailand[b] ;

Centre for Tropical Medicine and Global Health, Nuffield Department of Medicine, University of Oxford, Oxford, United Kingdom[c] ;

Swiss Tropical and Public Health Institute, Basel, Switzerland[d] **;**

Faculty of Medicine, University Basel, Basel, Switzerland[e] ;

Pacific Biosciences, 1305 O'Brien Drive, Menlo Park, CA 94025, USA[f] ;

Department of Statistics, University of Oxford, Oxford, OX1 3TG, UK[g]

Department of Medicine, Division of Infectious Diseases, Yong Loo Lin School of Medicine, National University of Singapore, Singapore[h]

Genome Institute of Singapore, A*STAR, Singapore 138672[i]

US Naval Medicine Research Center, Silver Spring, Maryland, USA[j]

[#] Address correspondence to Jeanne Salje: jeanne.salje@ndm.ox.ac.uk

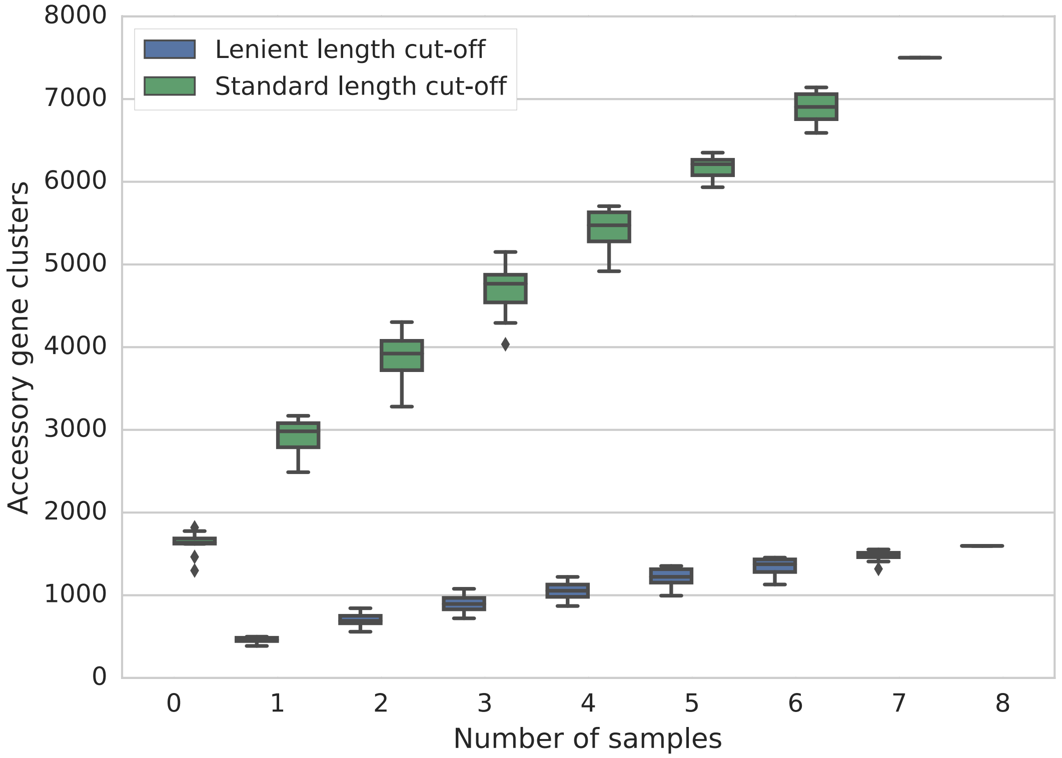

Figure S1. Boxplot showing how the number of accessory genes clusters varies with number of samples using the standard length cut-off and a more lenient length cut-off when determining clusters. Using the same identity thresholds but allowing the genes to be shorter reduces the number of new clusters identified as samples are added.

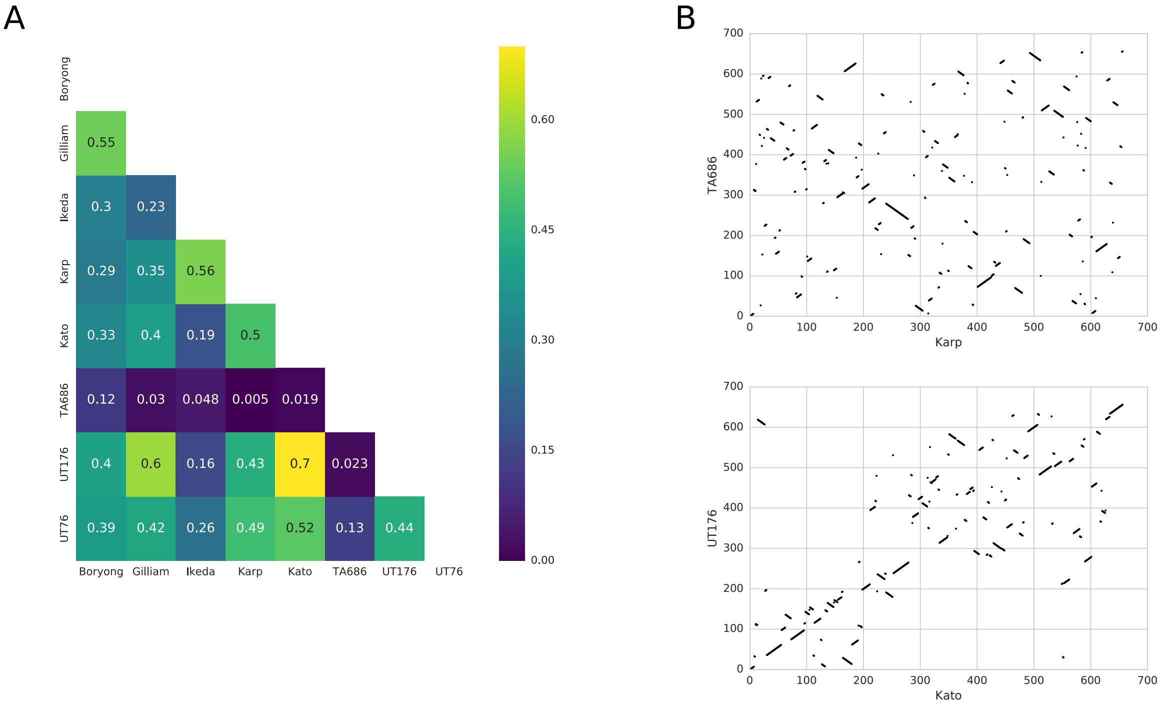

Figure S2. A - Heatmap showing the correlation in gene order between each pair of samples. B – dotplots showing the gene ordering between the pair with the highest correlation (Kato and UT176) and the lowest correlation (Karp and TA686).

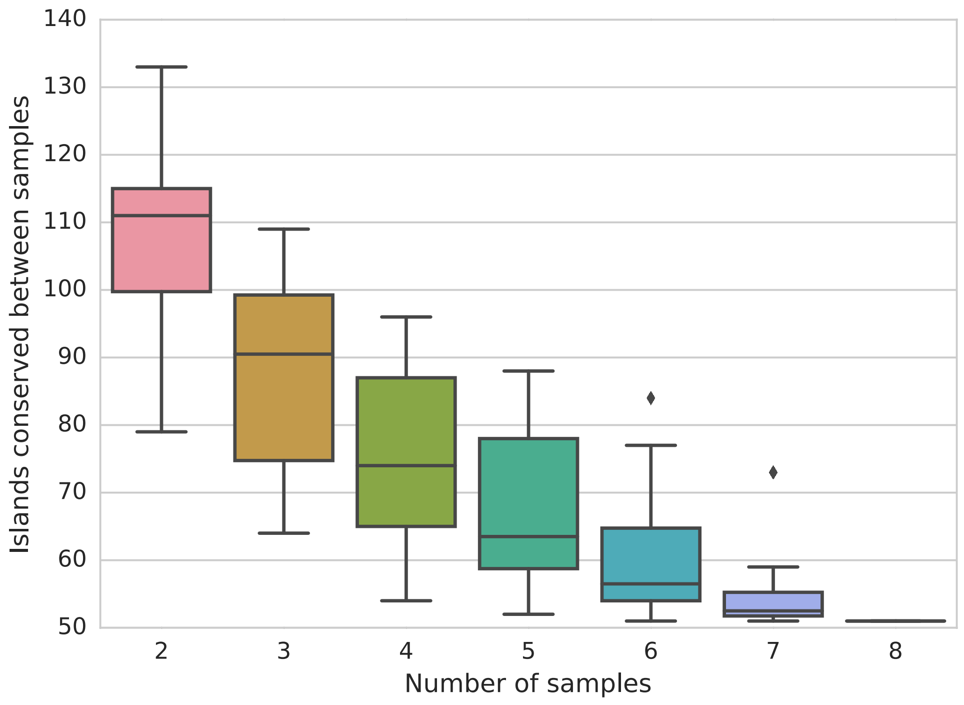

Figure S3. Boxplot showing the number of islands conserved between samples across all different combinations of samples.

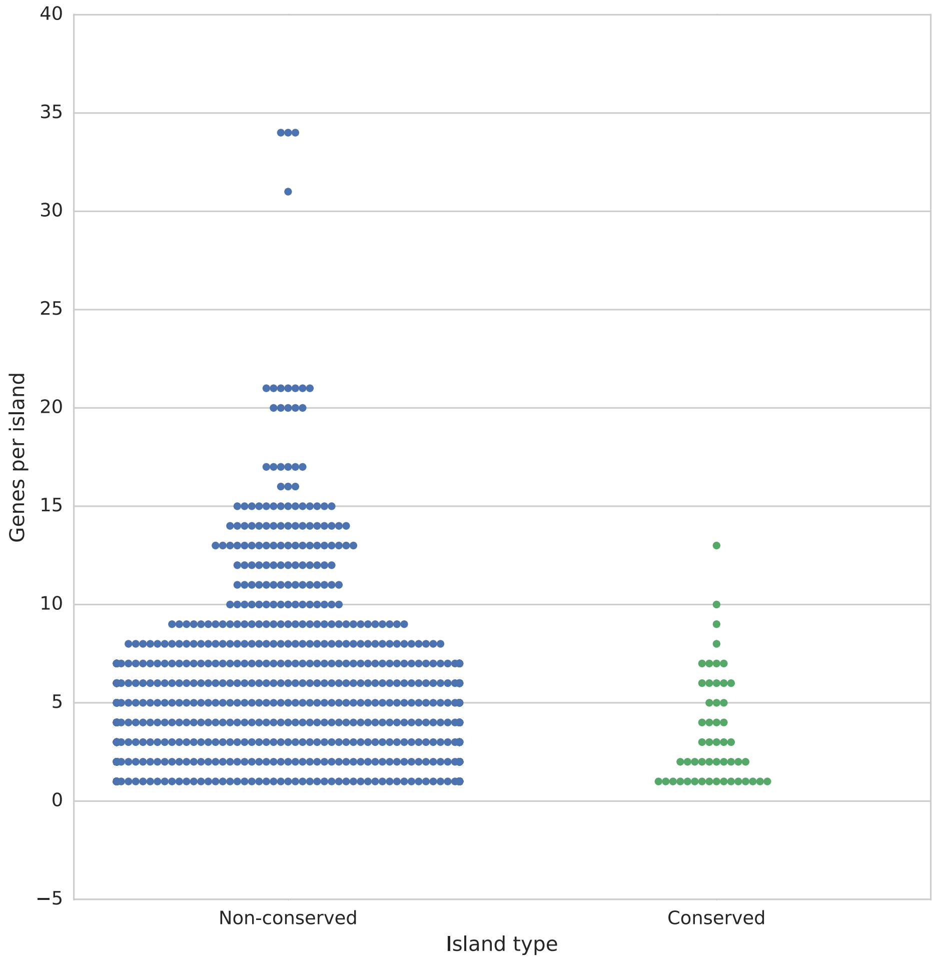

Figure S4. The number of genes per island in conserved versus non-conserved islands.

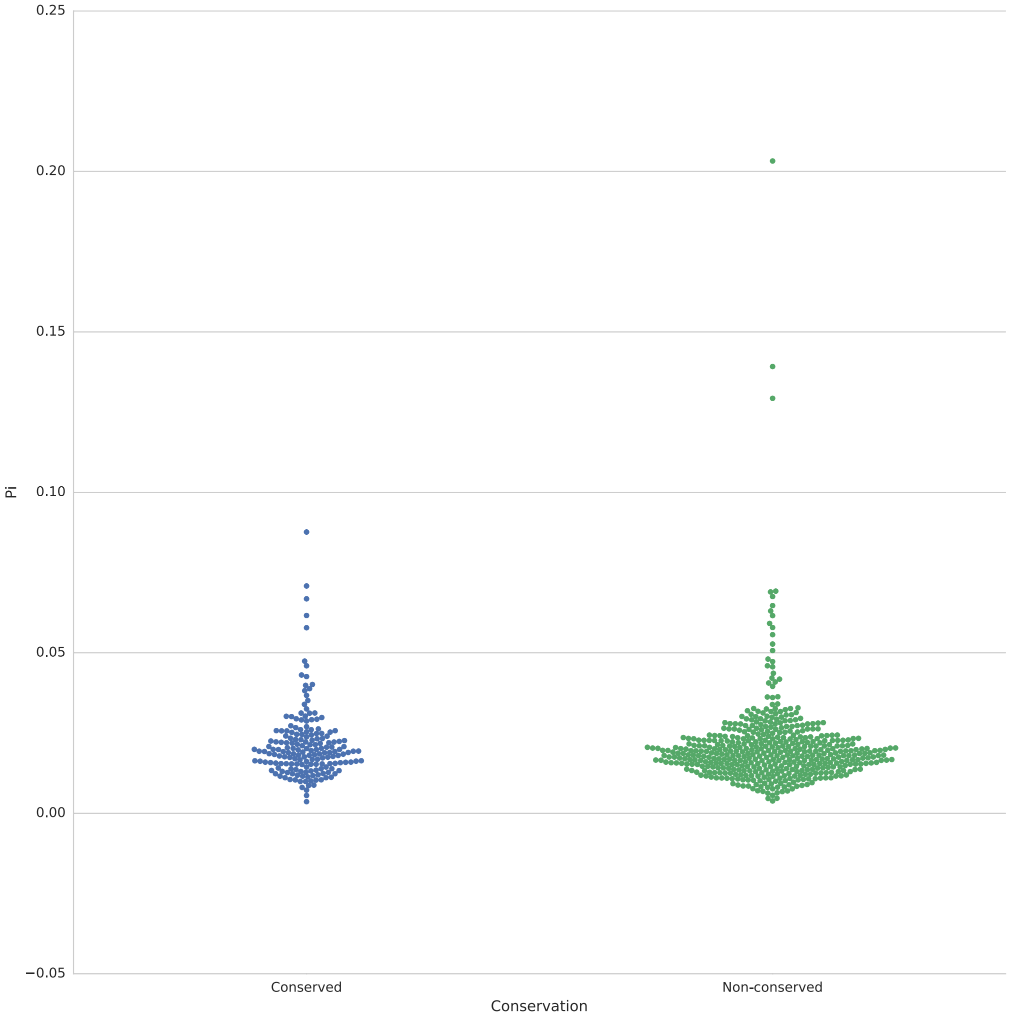

Figure S5. The nucleotide sequence diversity (π) in genes in conserved and non-conserved islands.

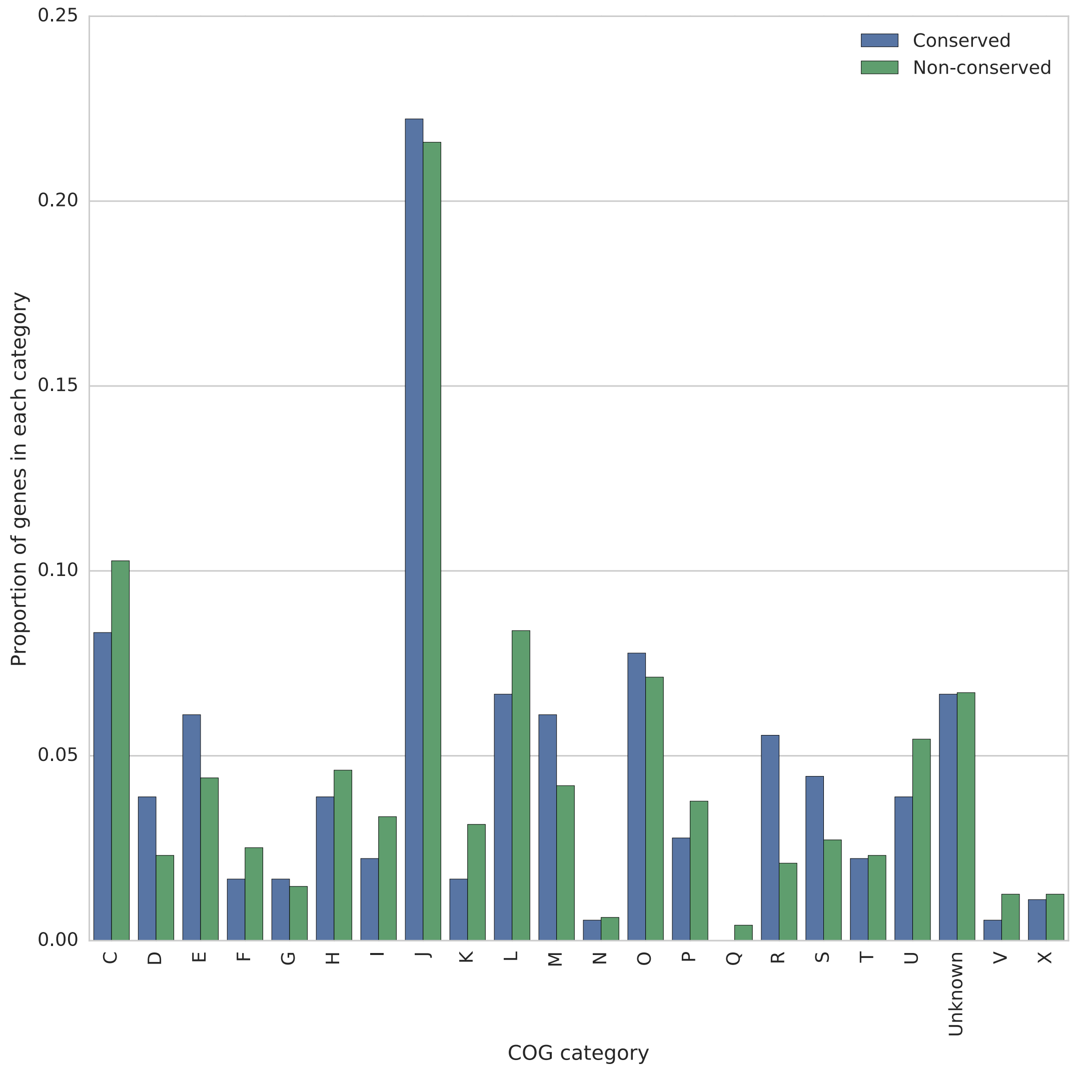

Figure S6. The proportion of core genes which are in conserved and non-conserved islands in each COG category.

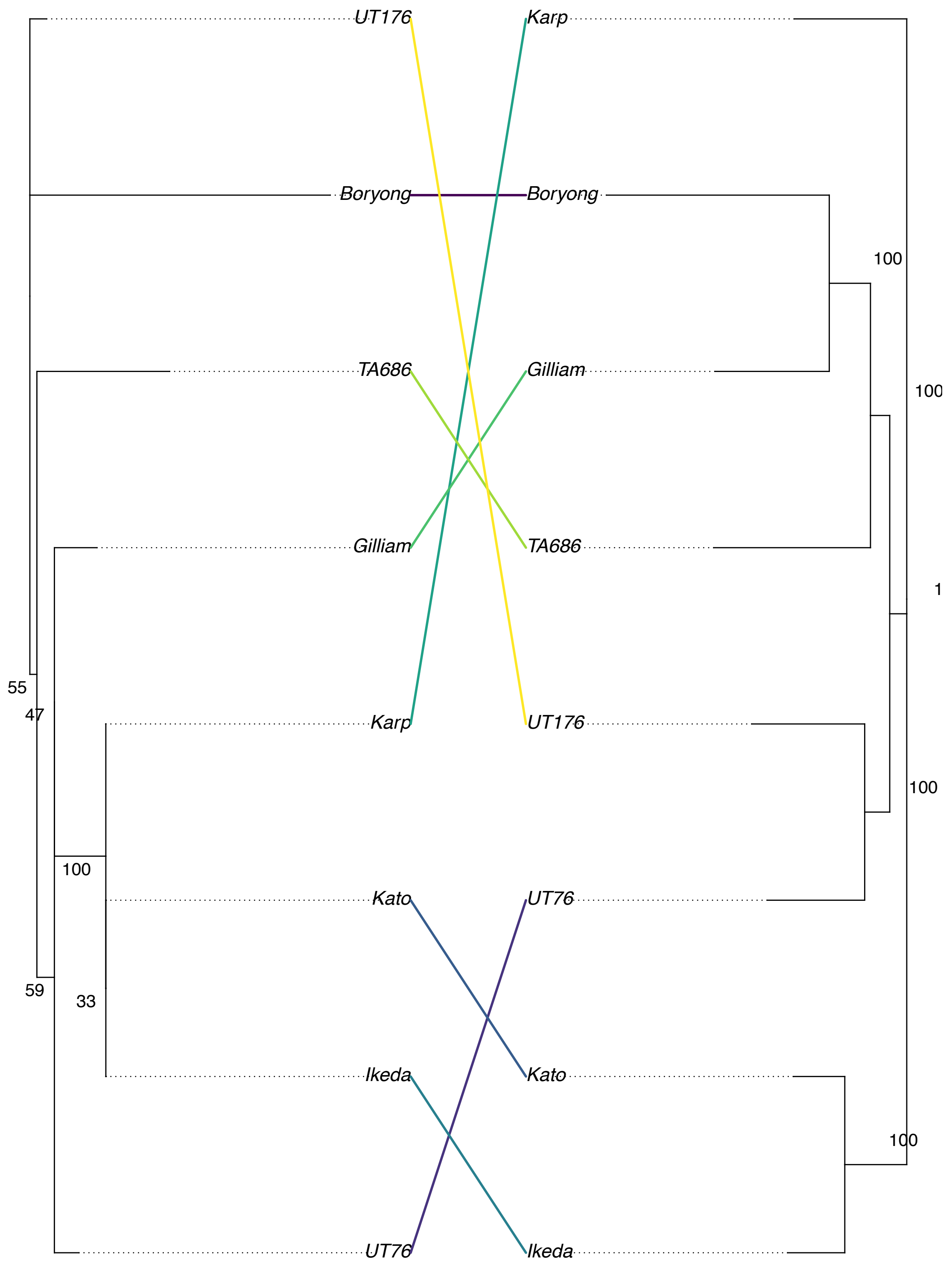

Figure S7. A phylogenetic tree showing the relationship between a tree generated using the 47kDa antigen sequences, and the sequences of 657 core genes.

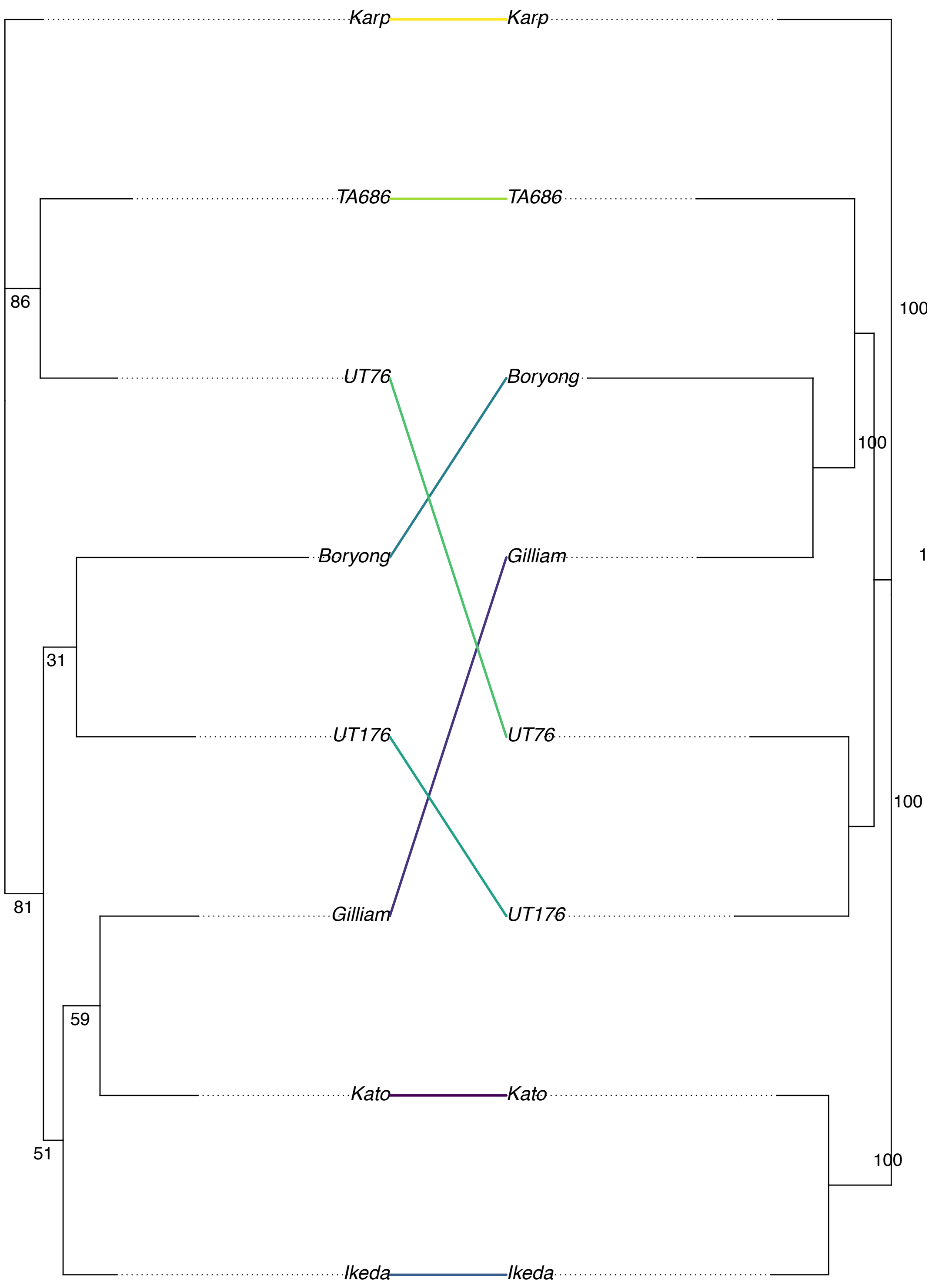

Figure S8. A phylogenetic tree showing the relationship between a tree generated using MLST gene sequences, and the sequences of 657 core genes. The 7 MLST genes (gpsA, mdh, nrdB, nuoF, ppdK, sucD, and sucB) are taken from the scheme developed by Sonthayanon et al., 2010.

Strain	Illumina sequencing	Illumina accession	Illumina read length	Illumina base pairs generated (Gbp)	Illumina coverage	PacBio accession	PacBio base pairs generated (Gbp)	PacBio number of reads	PacBio n50 read length	PacBio mean read length	PacBio coverage
Karp	Institute for Genome Sciences	PRJNA212440	300bp paired-end	3.15	1434	PRJEB24834	0.31	20,789	23,354	15,147	143
Kato	Institute for Genome Sciences	PRJNA212441	300bp paired-end	3.23	1469	PRJEB24834	1.35	75,987	28,132	17,782	614
Gilliam	Institute for Genome Sciences	PRJNA212442	300bp paired-end	3.30	1501	PRJEB24834	1.37	81,890	40,494	16,678	621
TA686	MicrobesNG	PRJEB24834	250bp paired-end	1.39	63	PRJEB24834	0.35	23,153	22,742	15,013	158
TA763	Institute for Genome Sciences	PRJNA212454	300bp paired-end	4.30	1954	PRJEB24834	2.10	84,163	41,496	24,912	953
FPW1038	Oxford Genomics Centre	PRJEB24834	300bp paired-end	1.63	742	PRJEB24834	2.37	95,037	40,662	24,910	1076
UT76	Oxford Genomics Centre	PRJEB24834	300bp paired-end	0.78	357	PRJEB24834	0.69	46,978	28,716	14,698	314
UT176	Oxford Genomics Centre	PRJEB24834	300bp paired-end	2,286,026,448	1039	PRJEB24834	647,714,986	45,432	20,728	14,256	294

Table S1. Data sources and sequencing statistics for the Illumina and PacBio sequencing data used in this study. Coverage has been estimated based on the base pairs of sequence data generated divided by the length of the 2.2Mb reference genome. All PacBio data was generated by the Genome Institute of Singapore.

Genome	NCBI Identifier
Orientia tsutsugamushi strain Boryong	GCF_000063545.1
Orientia tsutsugamushi strain Ikeda	GCF_000010205.1
Rickettsia typhi strain Wilmington	GCF_000008045.1
Rickettsia endosymbiont of *Ixodes scapularis*	GCF_000160735.1

Table S2. NCBI identifiers for previously published strains used in this paper.

Sample	Genome Length	Length of core genes	Core genes as proportion of genome	Length of repeat genes	Repeat genes as percentage of genome
Boryong	2127051	679631	0.32	748541	35
Gilliam	2465012	681491	0.28	1165831	47
Ikeda	2008987	683889	0.34	757868	38
Karp	2469803	682061	0.28	1163785	47
Kato	2319449	682142	0.29	1039243	45
TA686	2254553	682706	0.30	933469	41
UT176	1932116	681689	0.35	738572	38
UT76	2078193	682964	0.33	826716	40

Table S3. Core gene and core repeat statistics.

Table S4. Core genes calculated by Roary. Gene names are given for the Karp strain.

Gene group	Group number	Annotation	Gene name	Boryong	Gilliam	Ikeda	Karp	Kato	TA686	UT176	UT76-HP
clpP	1	ATP-dependent Clp protease proteolytic subunit		Boryong_01567	Gilliam_01942	Ikeda_00423	Karp_01574	Kato_01535	TA686_02079	UT176_01755	UT76-HP_01648
gatB	2	aspartyl/glutamyl-tRNA(Asn/Gln) amidotransferase subunit B	GatB	Boryong_01584	Gilliam_01957	Ikeda_00409	Karp_01279	Kato_01521	TA686_00288	UT176_01741	UT76-HP_01661
gatA	2	glutamyl-tRNA(Gln) amidotransferase subunit A	GatA	Boryong_01583	Gilliam_01956	Ikeda_00410	Karp_01280	Kato_01522	TA686_00289	UT176_01742	UT76-HP_01660
group_5707	2	aspartyl/glutamyl-tRNA(Asn/Gln) amidotransferase subunit C	GatC	Boryong_01582	Gilliam_01955	Ikeda_00411	Karp_01281	Kato_01523	TA686_00290	UT176_01743	UT76-HP_01659
group_6080	2	RNase J family beta-CASP ribonuclease		Boryong_01581	Gilliam_01954	Ikeda_00412	Karp_01282	Kato_01524	TA686_00291	UT176_01744	UT76-HP_01658
group_7975	2	DNA-binding response regulator		Boryong_01580	Gilliam_01953	Ikeda_00413	Karp_01283	Kato_01525	TA686_00292	UT176_01745	UT76-HP_01657
group_250	3	transposase		Boryong_00790	Gilliam_02703	Ikeda_02118	Karp_00040	Kato_00709	TA686_01162	UT176_00574	UT76-HP_00998
group_5845	3	multidrug ABC transporter ATP-binding protein		Boryong_00791	Gilliam_02704	Ikeda_02119	Karp_00041	Kato_00710	TA686_01161	UT176_00573	UT76-HP_00999
group_7831	3	UMP kinase		Boryong_00792	Gilliam_02705	Ikeda_02120	Karp_00042	Kato_00711	TA686_01160	UT176_00572	UT76-HP_01000
group_5846	3	phospho-N-acetylmuramoyl-pentapeptide- transferase	MraY	Boryong_00793	Gilliam_02706	Ikeda_02121	Karp_00043	Kato_00712	TA686_01159	UT176_00571	UT76-HP_01001
group_5550	3	UDP-N-acetylmuramoylalanyl-D-glutamyl-2, 6-diaminopimelate--D-alanyl-D-alanine ligase	MurF	Boryong_00794	Gilliam_02707	Ikeda_02122	Karp_00044	Kato_00713	TA686_01158	UT176_00570	UT76-HP_01002
group_5397	3	UDP-N-acetylmuramoyl-L-alanyl-D-glutamate--2, 6-diaminopimelate ligase	MurE	Boryong_00795	Gilliam_02708	Ikeda_02123	Karp_00045	Kato_00714	TA686_01157	UT176_00569	UT76-HP_01003
group_5847	3	penicillin-binding protein	PBP	Boryong_00796	Gilliam_02709	Ikeda_02124	Karp_00046	Kato_00715	TA686_01156	UT176_00568	UT76-HP_01004
ftsL	3	hypothetical protein	FtsL	Boryong_00797	Gilliam_02710	Ikeda_02125	Karp_00047	Kato_00716	TA686_01155	UT176_00567	UT76-HP_01005
group_5670	3	16S rRNA methyltransferase		Boryong_00798	Gilliam_02711	Ikeda_02126	Karp_00048	Kato_00717	TA686_01154	UT176_00566	UT76-HP_01006
group_6027	3	molecular chaperone DnaJ	DnaJ	Boryong_00799	Gilliam_02712	Ikeda_02127	Karp_00049	Kato_00718	TA686_01153	UT176_00565	UT76-HP_01007
group_7111	3	molecular chaperone DnaK	DnaK	Boryong_00800	Gilliam_02713	Ikeda_02128	Karp_00050	Kato_00719	TA686_01152	UT176_00564	UT76-HP_01008
group_6028	3	BolA family transcriptional regulator		Boryong_00801	Gilliam_02714	Ikeda_02129	Karp_00051	Kato_00720	TA686_01151	UT176_00563	UT76-HP_01009
group_5671	3	enoyl-ACP reductase	ENR	Boryong_00802	Gilliam_02715	Ikeda_02130	Karp_00052	Kato_00721	TA686_01150	UT176_00562	UT76-HP_01010
group_4752	4	sodium:proline symporter		Boryong_00980	Gilliam_00683	Ikeda_01863	Karp_00697	Kato_02375	TA686_02102	UT176_02068	UT76-HP_02241
group_5324	5	hypothetical protein		Boryong_00010	Gilliam_00014	Ikeda_01778	Karp_00009	Kato_00009	TA686_01198	UT176_00009	UT76-HP_00009
group_5345	6	hypothetical protein		Boryong_00676	Gilliam_02258	Ikeda_01084	Karp_02002	Kato_00906	TA686_02341	UT176_00940	UT76-HP_01866
group_5664	6	UDP-N-acetylmuramate--L-alanine ligase	MurC	Boryong_00675	Gilliam_02259	Ikeda_01085	Karp_02003	Kato_00905	TA686_02340	UT176_00939	UT76-HP_01867
group_5663	6	UDP-N-acetylenolpyruvoylglucosamine reductase	MurB	Boryong_00674	Gilliam_02260	Ikeda_01086	Karp_02004	Kato_00904	TA686_02339	UT176_00938	UT76-HP_01868
group_5839	6	D-alanine--D-alanine ligase	Ddl	Boryong_00673	Gilliam_02261	Ikeda_01087	Karp_02005	Kato_00903	TA686_02338	UT176_00937	UT76-HP_01869
group_5662	6	cell division protein FtsQ	FtsQ	Boryong_00672	Gilliam_02262	Ikeda_01088	Karp_02006	Kato_00902	TA686_02337	UT176_00936	UT76-HP_01870
group_5548	6	DNA replication/repair protein RecF	RecF	Boryong_00671	Gilliam_02263	Ikeda_01089	Karp_02007	Kato_00901	TA686_02336	UT176_00935	UT76-HP_01871
group_5349	7	hypothetical protein		Boryong_01099	Gilliam_00969	Ikeda_00001	Karp_02387	Kato_02127	TA686_01419	UT176_00668	UT76-HP_00144
group_6051	7	virB4 protein precursor		Boryong_01098	Gilliam_00970	Ikeda_00002	Karp_02388	Kato_02126	TA686_01418	UT176_00667	UT76-HP_00143
group_5858	7	type I glyceraldehyde-3-phosphate dehydrogenase	GapA	Boryong_01097	Gilliam_00971	Ikeda_00003	Karp_02389	Kato_02125	TA686_01417	UT176_00666	UT76-HP_00142
group_5857	7	phosphoglycerate kinase	Pgk	Boryong_01096	Gilliam_00972	Ikeda_00004	Karp_02390	Kato_02124	TA686_01416	UT176_00665	UT76-HP_00141
group_6050	7	hypothetical protein		Boryong_01095	Gilliam_00973	Ikeda_00005	Karp_02391	Kato_02123	TA686_01415	UT176_00664	UT76-HP_00140
group_5856	7	proline--tRNA ligase	ProS	Boryong_01094	Gilliam_00974	Ikeda_00006	Karp_02392	Kato_02122	TA686_01414	UT176_00663	UT76-HP_00139

group_7234	7	ATP-dependent Clp protease ATP-binding subunit ClpX	ClpX	Boryong_01093	Gilliam_00975	Ikeda_00007	Karp_02393	Kato_02121	TA686_01413	UT176_00662	UT76-HP_00138
group_8077	7	elongation factor P		Boryong_01092	Gilliam_00976	Ikeda_00008	Karp_02394	Kato_02120	TA686_01412	UT176_00661	UT76-HP_00137
group_6049	7	extragenic suppressor protein SuhB	SuhB	Boryong_01091	Gilliam_00977	Ikeda_00009	Karp_02395	Kato_02119	TA686_01411	UT176_00660	UT76-HP_00136
group_5557	7	tRNA (adenosine(37)-N6)-threonylcarbamoyltransferase complex dimerization subunit type 1 TsaB	TsaB	Boryong_01090	Gilliam_00978	Ikeda_00010	Karp_02396	Kato_02118	TA686_01410	UT176_00659	UT76-HP_00135
group_5351	8	hypothetical protein		Boryong_01511	Gilliam_01339	Ikeda_01143	Karp_01848	Kato_00841	TA686_00231	UT176_01364	UT76-HP_01169
group_5352	9	glycerol-3-phosphate dehydrogenase (NAD(P)( ))	GpsA	Boryong_01513	Gilliam_01337	Ikeda_01145	Karp_01850	Kato_00843	TA686_00229	UT176_01366	UT76-HP_01171
group_8119	9	tRNA (N(6)-L-threonylcarbamoyladenosine(37)-C(2))-methylthiotransferase MtaB	MtaB	Boryong_01514	Gilliam_01336	Ikeda_01146	Karp_01851	Kato_00844	TA686_00228	UT176_01367	UT76-HP_01172
group_5359	10	crossover junction endodeoxyribonuclease RuvC	RuvC	Boryong_01866	Gilliam_01437	Ikeda_01053	Karp_02233	Kato_00938	TA686_02092	UT176_01131	UT76-HP_01610
group_5717	10	tRNA dihydrouridine synthase DusB	DusB	Boryong_01867	Gilliam_01438	Ikeda_01054	Karp_02234	Kato_00937	TA686_02091	UT176_01132	UT76-HP_01611
group_5494	10	hypothetical protein		Boryong_01868	Gilliam_01439	Ikeda_01055	Karp_02235	Kato_00936	TA686_02090	UT176_01133	UT76-HP_01612
group_5495	10	bifunctional 3-demethylubiquinone 3-O-methyltransferase/2-octaprenyl-6-hydroxy phenol methylase		Boryong_01869	Gilliam_01440	Ikeda_01056	Karp_02236	Kato_00935	TA686_02089	UT176_01134	UT76-HP_01613
group_5718	10	protein-(glutamine-N5) methyltransferase, release factor-specific		Boryong_01870	Gilliam_01441	Ikeda_01057	Karp_02237	Kato_00934	TA686_02088	UT176_01135	UT76-HP_01614
group_6104	10	tRNA pseudouridine(38-40) synthase TruA	TruA	Boryong_01871	Gilliam_01442	Ikeda_01058	Karp_02238	Kato_00933	TA686_02087	UT176_01136	UT76-HP_01615
group_7746	10	50S ribosomal protein L13	L13	Boryong_01872	Gilliam_01443	Ikeda_01059	Karp_02239	Kato_00932	TA686_02086	UT176_01137	UT76-HP_01616
group_5719	10	30S ribosomal protein S9	S9	Boryong_01873	Gilliam_01444	Ikeda_01060	Karp_02240	Kato_00931	TA686_02085	UT176_01138	UT76-HP_01617
group_5458	11	rRNA (cytidine-2'-O-)-methyltransferase		Boryong_01202	Gilliam_01733	Ikeda_00482	Karp_01858	Kato_01661	TA686_00545	UT176_01357	UT76-HP_01640
group_5865	11	serine--tRNA ligase	SerS	Boryong_01203	Gilliam_01734	Ikeda_00481	Karp_01859	Kato_01662	TA686_00546	UT176_01356	UT76-HP_01639
group_7705	11	twin-arginine translocase subunit TatC	TatC	Boryong_01204	Gilliam_01735	Ikeda_00480	Karp_01860	Kato_01663	TA686_00547	UT176_01355	UT76-HP_01638
group_6566	11	hypothetical protein		Boryong_01205	Gilliam_01736	Ikeda_00479	Karp_01861	Kato_01664	TA686_00548	UT176_01354	UT76-HP_01637
group_6058	11	16S rRNA methyltransferase		Boryong_01206	Gilliam_01737	Ikeda_00478	Karp_01862	Kato_01665	TA686_00549	UT176_01353	UT76-HP_01636
group_7851	11	chromosome partitioning protein ParA	ParA	Boryong_01207	Gilliam_01738	Ikeda_00477	Karp_01863	Kato_01666	TA686_00550	UT176_01352	UT76-HP_01635
group_6059	11	chromosome partitioning protein	ParB	Boryong_01208	Gilliam_01739	Ikeda_00476	Karp_01864	Kato_01667	TA686_00551	UT176_01351	UT76-HP_01634
group_5485	12	rod shape-determining protein MreC	MreC	Boryong_01561	Gilliam_02176	Ikeda_00336	Karp_01810	Kato_01222	TA686_01169	UT176_01839	UT76-HP_01300
group_7287	12	rod shape-determining protein	MreB	Boryong_01562	Gilliam_02175	Ikeda_00335	Karp_01811	Kato_01223	TA686_01168	UT176_01838	UT76-HP_01301
group_5575	12	dihydrolipoamide acetyltransferase		Boryong_01563	Gilliam_02174	Ikeda_00334	Karp_01812	Kato_01224	TA686_01167	UT176_01837	UT76-HP_01302
group_5491	13	aspartate kinase	AK	Boryong_01771	Gilliam_01906	Ikeda_00772	Karp_01348	Kato_01421	TA686_00345	UT176_01725	UT76-HP_01353
group_5712	13	hypothetical protein		Boryong_01772	Gilliam_01905	Ikeda_00773	Karp_01349	Kato_01420	TA686_00346	UT176_01724	UT76-HP_01354
group_8049	13	potassium transporter		Boryong_01773	Gilliam_01904	Ikeda_00774	Karp_01350	Kato_01419	TA686_00347	UT176_01723	UT76-HP_01355
group_5713	13	5-formyltetrahydrofolate cyclo-ligase	YgfA	Boryong_01774	Gilliam_01903	Ikeda_00775	Karp_01351	Kato_01418	TA686_00348	UT176_01722	UT76-HP_01356
group_5579	13	hypothetical protein		Boryong_01775	Gilliam_01902	Ikeda_00776	Karp_01352	Kato_01417	TA686_00349	UT176_01721	UT76-HP_01357
group_5496	14	ankyrin repeat-containing protein 13	Ank13	Boryong_01925	Gilliam_01541	Ikeda_00523	Karp_01630	Kato_01772	TA686_00095	UT176_01272	UT76-HP_01784
group_5411	14	hypothetical protein		Boryong_01926	Gilliam_01542	Ikeda_00522	Karp_01631	Kato_01773	TA686_00094	UT176_01273	UT76-HP_01785
group_5497	15	heme A synthase		Boryong_02136	Gilliam_01573	Ikeda_00787	Karp_01402	Kato_01407	TA686_01213	UT176_01711	UT76-HP_01347

group_5549	16	threonylcarbamoyl-AMP synthase	TsaC	Boryong_00680	Gilliam_02254	Ikeda_01080	Karp_01998	Kato_00910	TA686_02345	UT176_00944	UT76-HP_01862
group_5448	16	glycine--tRNA ligase subunit beta	GlyS	Boryong_00679	Gilliam_02255	Ikeda_01081	Karp_01999	Kato_00909	TA686_02344	UT176_00943	UT76-HP_01863
group_5840	16	glycine--tRNA ligase subunit alpha	GlyQ	Boryong_00678	Gilliam_02256	Ikeda_01082	Karp_02000	Kato_00908	TA686_02343	UT176_00942	UT76-HP_01864
group_5574	17	competence protein ComEC	ComEC	Boryong_01456	Gilliam_02183	Ikeda_00344	Karp_01804	Kato_01216	TA686_00950	UT176_01848	UT76-HP_01293
group_5585	18	hypothetical protein		Boryong_01875	Gilliam_01446	Ikeda_01062	Karp_02242	Kato_00929	TA686_02083	UT176_01140	UT76-HP_01619
group_5622	19	hypothetical protein		Boryong_00133	Gilliam_01746	Ikeda_01832	Karp_00722	Kato_02398	TA686_01802	UT176_02092	UT76-HP_02218
group_5776	19	2,3,4,5-tetrahydropyridine-2,6-dicarboxylate N-succinyltransferase	DapD	Boryong_00132	Gilliam_01745	Ikeda_01831	Karp_00723	Kato_02399	TA686_01803	UT176_02093	UT76-HP_02217
group_5656	20	MFS transporter permease		Boryong_00574	Gilliam_00985	Ikeda_01615	Karp_00351	Kato_00491	TA686_00383	UT176_00185	UT76-HP_00366
group_5544	20	sodium:pantothenate symporter		Boryong_00573	Gilliam_00986	Ikeda_01616	Karp_00352	Kato_00492	TA686_00382	UT176_00186	UT76-HP_00367
group_5684	21	SAM-dependent methyltransferase		Boryong_00940	Gilliam_02522	Ikeda_01914	Karp_00101	Kato_02345	TA686_00107	UT176_00553	UT76-HP_01014
group_5705	22	two-component sensor histidine kinase		Boryong_01454	Gilliam_02181	Ikeda_00342	Karp_01806	Kato_01218	TA686_00952	UT176_01846	UT76-HP_01295
group_6160	22	sigma-54-dependent Fis family transcriptional regulator		Boryong_01453	Gilliam_02180	Ikeda_00341	Karp_01807	Kato_01219	TA686_00953	UT176_01845	UT76-HP_01296
group_5483	22	hypothetical protein		Boryong_01452	Gilliam_02179	Ikeda_00340	Karp_01808	Kato_01220	TA686_00954	UT176_01844	UT76-HP_01297
group_5722	23	aspartate aminotransferase	AspC	Boryong_02006	Gilliam_00094	Ikeda_01340	Karp_00229	Kato_00160	TA686_02116	UT176_00101	UT76-HP_00570
ubiG	23	Ubiquinone biosynthesis O-methyltransferase	UbiG	Boryong_02007	Gilliam_00095	Ikeda_01341	Karp_00230	Kato_00161	TA686_02115	UT176_00102	UT76-HP_00569
group_5723	23	ABC transporter		Boryong_02008	Gilliam_00096	Ikeda_01342	Karp_00231	Kato_00162	TA686_02114	UT176_00103	UT76-HP_00568
group_5724	23	hypothetical protein		Boryong_02009	Gilliam_00097	Ikeda_01343	Karp_00232	Kato_00163	TA686_02113	UT176_00104	UT76-HP_00567
group_5900	23	coproporphyrinogen III oxidase		Boryong_02010	Gilliam_00098	Ikeda_01344	Karp_00233	Kato_00164	TA686_02112	UT176_00105	UT76-HP_00566
group_6112	23	hypothetical protein		Boryong_02011	Gilliam_00099	Ikeda_01345	Karp_00234	Kato_00165	TA686_02111	UT176_00106	UT76-HP_00565
group_5587	23	DNA repair protein RecO	RecO	Boryong_02012	Gilliam_00100	Ikeda_01346	Karp_00235	Kato_00166	TA686_02110	UT176_00107	UT76-HP_00564
group_5732	24	DNA helicase II	UvrD	Boryong_02217	Gilliam_00618	Ikeda_00573	Karp_01153	Kato_01479	TA686_01997	UT176_01493	UT76-HP_01067
group_5740	25	NAD-glutamate dehydrogenase	GdhA	Boryong_02452	Gilliam_01083	Ikeda_02116	Karp_02529	Kato_00707	TA686_01765	UT176_00639	UT76-HP_00743
group_5739	25	tRNA uridine-5-carboxymethylaminomethyl(34) synthesis GTPase MnmE		Boryong_02451	Gilliam_01084	Ikeda_02115	Karp_02530	Kato_00706	TA686_01766	UT176_00640	UT76-HP_00744
group_6137	25	recombinase XerC	XerC	Boryong_02450	Gilliam_01085	Ikeda_02114	Karp_02531	Kato_00705	TA686_01767	UT176_00641	UT76-HP_00745
group_6136	25	RNA polymerase-binding protein DksA	DksA	Boryong_02449	Gilliam_01086	Ikeda_02113	Karp_02532	Kato_00704	TA686_01768	UT176_00642	UT76-HP_00746
group_6135	25	inorganic pyrophosphatase		Boryong_02448	Gilliam_01087	Ikeda_02112	Karp_02533	Kato_00703	TA686_01769	UT176_00643	UT76-HP_00747
group_5415	25	DNA polymerase III subunit delta'	HolB	Boryong_02447	Gilliam_01088	Ikeda_02111	Karp_02534	Kato_00702	TA686_01770	UT176_00644	UT76-HP_00748
group_5922	25	ribosomal large subunit pseudouridine synthase		Boryong_02446	Gilliam_01089	Ikeda_02110	Karp_02535	Kato_00701	TA686_01771	UT176_00645	UT76-HP_00749
group_5791	26	tetraacyldisaccharide 4'-kinase	IpxK	Boryong_00218	Gilliam_01219	Ikeda_02020	Karp_00613	Kato_00657	TA686_00253	UT176_00427	UT76-HP_02120
group_7640	26	hypothetical protein		Boryong_00219	Gilliam_01220	Ikeda_02021	Karp_00614	Kato_00658	TA686_00254	UT176_00428	UT76-HP_02119
group_5849	27	transporter		Boryong_00935	Gilliam_02519	Ikeda_01910	Karp_00104	Kato_02349	TA686_01643	UT176_00549	UT76-HP_01018
glpE	27	hypothetical protein		Boryong_00934	Gilliam_02518	Ikeda_01909	Karp_00105	Kato_02350	TA686_01642	UT176_00548	UT76-HP_01019
group_5864	28	protein translocase subunit SecF	SecF	Boryong_01198	Gilliam_01729	Ikeda_00486	Karp_01854	Kato_01657	TA686_00541	UT176_01361	UT76-HP_01644
group_6056	28	DNA mismatch repair protein MutS	MutS	Boryong_01199	Gilliam_01730	Ikeda_00485	Karp_01855	Kato_01658	TA686_00542	UT176_01360	UT76-HP_01643

group_6057	28	ATP/ADP translocase		Boryong_01200	Gilliam_01731	Ikeda_00484	Karp_01856	Kato_01659	TA686_00543	UT176_01359	UT76-HP_01642
group_5882	29	haloacid dehalogenase		Boryong_01633	Gilliam_01659	Ikeda_00944	Karp_01021	Kato_01991	TA686_00732	UT176_01408	UT76-HP_00989
group_7708	29	DNA gyrase subunit B	GyrB	Boryong_01632	Gilliam_01658	Ikeda_00945	Karp_01022	Kato_01992	TA686_00733	UT176_01407	UT76-HP_00988
group_5577	29	hypothetical protein		Boryong_01631	Gilliam_01657	Ikeda_00946	Karp_01023	Kato_01993	TA686_00734	UT176_01406	UT76-HP_00987
group_5881	29	amino acid permease		Boryong_01630	Gilliam_01656	Ikeda_00947	Karp_01024	Kato_01994	TA686_00735	UT176_01405	UT76-HP_00986
group_5895	30	succinate dehydrogenase iron-sulfur subunit	SdhB	Boryong_01938	Gilliam_00945	Ikeda_02208	Karp_02372	Kato_02140	TA686_00235	UT176_00689	UT76-HP_00157
group_6106	30	succinate dehydrogenase flavoprotein subunit	SdhA	Boryong_01937	Gilliam_00944	Ikeda_02209	Karp_02373	Kato_02139	TA686_00236	UT176_00688	UT76-HP_00156
group_5586	30	succinate dehydrogenase, hydrophobic membrane anchor protein	SdhD	Boryong_01936	Gilliam_00943	Ikeda_02210	Karp_02374	Kato_02138	TA686_00237	UT176_00687	UT76-HP_00155
group_5721	30	succinate dehydrogenase, cytochrome b556 subunit	SdhC	Boryong_01935	Gilliam_00942	Ikeda_02211	Karp_02375	Kato_02137	TA686_00238	UT176_00686	UT76-HP_00154
group_5901	31	hypothetical protein		Boryong_02018	Gilliam_00108	Ikeda_01353	Karp_00239	Kato_00172	TA686_01204	UT176_00113	UT76-HP_00558
group_6113	31	hypothetical protein		Boryong_02019	Gilliam_00109	Ikeda_01354	Karp_00240	Kato_00173	TA686_01205	UT176_00114	UT76-HP_00557
group_8000	31	30S ribosomal protein S12	RpsL	Boryong_02020	Gilliam_00110	Ikeda_01355	Karp_00241	Kato_00174	TA686_01206	UT176_00115	UT76-HP_00556
group_7893	31	30S ribosomal protein S7	RpsG	Boryong_02021	Gilliam_00111	Ikeda_01356	Karp_00242	Kato_00175	TA686_01207	UT176_00116	UT76-HP_00555
group_7759	31	elongation factor G	EfG	Boryong_02022	Gilliam_00112	Ikeda_01357	Karp_00243	Kato_00176	TA686_01208	UT176_00117	UT76-HP_00554
group_5902	31	30S ribosomal protein S1	RpsA	Boryong_02023	Gilliam_00113	Ikeda_01358	Karp_00244	Kato_00177	TA686_01209	UT176_00118	UT76-HP_00553
group_6017	32	50S ribosomal protein L20	RplT	Boryong_00628	Gilliam_02347	Ikeda_00902	Karp_00926	Kato_01948	TA686_00833	UT176_01165	UT76-HP_00870
group_7830	32	50S ribosomal protein L35	RpmL	Boryong_00627	Gilliam_02348	Ikeda_00903	Karp_00927	Kato_01949	TA686_00834	UT176_01166	UT76-HP_00871
group_8150	32	molecular chaperone HtpG	HtpG	Boryong_00626	Gilliam_02349	Ikeda_00904	Karp_00928	Kato_01950	TA686_00835	UT176_01167	UT76-HP_00872
group_5657	32	succinyl-diaminopimelate desuccinylase	DapE	Boryong_00625	Gilliam_02350	Ikeda_00905	Karp_00929	Kato_01951	TA686_00836	UT176_01168	UT76-HP_00873
group_6033	33	DNA translocase FtsK	FtsK	Boryong_00894	Gilliam_01256	Ikeda_00430	Karp_01269	Kato_01542	TA686_00583	UT176_01646	UT76-HP_01670
group_5682	33	hypothetical protein		Boryong_00895	Gilliam_01257	Ikeda_00429	Karp_01270	Kato_01541	TA686_00584	UT176_01647	UT76-HP_01669
group_7304	33	energy-dependent translational throttle protein EttA	EttA	Boryong_00896	Gilliam_01258	Ikeda_00428	Karp_01271	Kato_01540	TA686_00585	UT176_01648	UT76-HP_01668
group_5348	33	hypothetical protein		Boryong_00897	Gilliam_01259	Ikeda_00427	Karp_01272	Kato_01539	TA686_00586	UT176_01649	UT76-HP_01667
group_6064	34	alpha/beta hydrolase		Boryong_01286	Gilliam_01621	Ikeda_00655	Karp_01105	Kato_01727	TA686_00811	UT176_01536	UT76-HP_00785
group_5701	34	iron-sulfur-binding protein		Boryong_01285	Gilliam_01620	Ikeda_00654	Karp_01106	Kato_01726	TA686_00812	UT176_01535	UT76-HP_00784
group_5400	34	aminotransferase class V-fold PLP-dependent enzyme		Boryong_01284	Gilliam_01619	Ikeda_00653	Karp_01107	Kato_01725	TA686_00813	UT176_01534	UT76-HP_00783
group_6063	34	cysteine desulfurase		Boryong_01283	Gilliam_01618	Ikeda_00652	Karp_01108	Kato_01724	TA686_00814	UT176_01533	UT76-HP_00782
group_5868	34	iron-sulfur cluster scaffold-like protein		Boryong_01282	Gilliam_01617	Ikeda_00651	Karp_01109	Kato_01723	TA686_00815	UT176_01532	UT76-HP_00781
group_6062	34	iron-sulfur cluster assembly accessory protein		Boryong_01281	Gilliam_01616	Ikeda_00650	Karp_01110	Kato_01722	TA686_00816	UT176_01531	UT76-HP_00780
group_5564	34	co-chaperone HscB	HscB	Boryong_01280	Gilliam_01615	Ikeda_00649	Karp_01111	Kato_01721	TA686_00817	UT176_01530	UT76-HP_00779
group_5867	34	molecular chaperone HscA	HscA	Boryong_01279	Gilliam_01614	Ikeda_00648	Karp_01112	Kato_01720	TA686_00818	UT176_01529	UT76-HP_00778
group_5563	34	(2Fe-2S) ferredoxin		Boryong_01278	Gilliam_01613	Ikeda_00647	Karp_01113	Kato_01719	TA686_00819	UT176_01528	UT76-HP_00777
group_6072	35	electron transporter		Boryong_01395	Gilliam_00196	Ikeda_01076	Karp_01763	Kato_00913	TA686_02251	UT176_01154	UT76-HP_01631

group_6074	36	single-stranded DNA-binding protein		Boryong_01420	Gilliam_01983	Ikeda_00830	Karp_01239	Kato_01364	TA686_01786	UT176_01802	UT76-HP_01743
group_5704	36	hypothetical protein		Boryong_01419	Gilliam_01982	Ikeda_00831	Karp_01240	Kato_01363	TA686_01785	UT176_01803	UT76-HP_01742
group_6078	37	malate dehydrogenase	Mdh	Boryong_01520	Gilliam_01526	Ikeda_00663	Karp_01409	Kato_01734	TA686_02541	UT176_01450	UT76-HP_01425
group_6077	37	permease		Boryong_01519	Gilliam_01527	Ikeda_00664	Karp_01410	Kato_01735	TA686_02540	UT176_01451	UT76-HP_01426
group_5484	37	hypothetical protein		Boryong_01518	Gilliam_01528	Ikeda_00665	Karp_01411	Kato_01736	TA686_02539	UT176_01452	UT76-HP_01427
group_6083	38	cytochrome b	CybB	Boryong_01614	Gilliam_02691	Ikeda_00819	Karp_01467	Kato_01374	TA686_00793	UT176_01660	UT76-HP_01121
group_5878	38	ubiquinol-cytochrome c reductase iron-sulfur subunit	PetA	Boryong_01613	Gilliam_02690	Ikeda_00820	Karp_01468	Kato_01373	TA686_00792	UT176_01661	UT76-HP_01120
group_5486	38	hypothetical protein		Boryong_01612	Gilliam_02689	Ikeda_00821	Karp_01469	Kato_01372	TA686_00791	UT176_01662	UT76-HP_01119
group_5877	38	heme exporter protein B	CcmB	Boryong_01611	Gilliam_02688	Ikeda_00822	Karp_01470	Kato_01371	TA686_00790	UT176_01663	UT76-HP_01118
group_5709	38	cytochrome c biogenesis protein CcmA	CcmA	Boryong_01610	Gilliam_02687	Ikeda_00823	Karp_01471	Kato_01370	TA686_00789	UT176_01664	UT76-HP_01117
group_6087	39	2-hydroxyacid dehydrogenase		Boryong_01640	Gilliam_01666	Ikeda_00937	Karp_01014	Kato_01984	TA686_00725	UT176_01415	UT76-HP_00996
group_7914	39	cation:proton antiporter		Boryong_01639	Gilliam_01665	Ikeda_00938	Karp_01015	Kato_01985	TA686_00726	UT176_01414	UT76-HP_00995
group_6086	39	cation:proton antiporter		Boryong_01638	Gilliam_01664	Ikeda_00939	Karp_01016	Kato_01986	TA686_00727	UT176_01413	UT76-HP_00994
group_5883	39	sodium:proton antiporter		Boryong_01637	Gilliam_01663	Ikeda_00940	Karp_01017	Kato_01987	TA686_00728	UT176_01412	UT76-HP_00993
group_5710	39	sodium:proton antiporter		Boryong_01636	Gilliam_01662	Ikeda_00941	Karp_01018	Kato_01988	TA686_00729	UT176_01411	UT76-HP_00992
group_8081	39	sodium:proton antiporter		Boryong_01635	Gilliam_01661	Ikeda_00942	Karp_01019	Kato_01989	TA686_00730	UT176_01410	UT76-HP_00991
group_6098	40	hypothetical protein		Boryong_01851	Gilliam_01422	Ikeda_01037	Karp_02217	Kato_00953	TA686_01496	UT176_01371	UT76-HP_01595
group_6107	41	S26 family signal peptidase		Boryong_01941	Gilliam_00952	Ikeda_02205	Karp_00806	Kato_02077	TA686_00272	UT176_00890	UT76-HP_02017
group_6108	41	ribonuclease III	Rnc	Boryong_01942	Gilliam_00953	Ikeda_02204	Karp_00807	Kato_02076	TA686_00273	UT176_00889	UT76-HP_02016
group_6121	42	nucleoside-diphosphate kinase	Ndk	Boryong_02109	Gilliam_00855	Ikeda_02149	Karp_02170	Kato_00743	TA686_01081	UT176_01940	UT76-HP_00186
group_6122	43	hypothetical protein		Boryong_02132	Gilliam_01567	Ikeda_00791	Karp_01397	Kato_01402	TA686_02328	UT176_01708	UT76-HP_01343
group_6132	44	phospholipase D family protein		Boryong_02222	Gilliam_00612	Ikeda_00567	Karp_01146	Kato_01485	TA686_01628	UT176_01487	UT76-HP_01062
group_6754	45	elongation factor 4	IepA	Boryong_01410	Gilliam_02108	Ikeda_00834	Karp_01242	Kato_01360	TA686_01368	UT176_01301	UT76-HP_01739
group_5874	45	peptide chain release factor 1	PrfA	Boryong_01409	Gilliam_02107	Ikeda_00835	Karp_01243	Kato_01359	TA686_01367	UT176_01302	UT76-HP_01738
group_7286	46	DNA-binding protein		Boryong_00490	Gilliam_00721	Ikeda_00027	Karp_02417	Kato_02102	TA686_00757	UT176_00621	UT76-HP_01986
surA	46	Chaperone SurA	SurA	Boryong_00489	Gilliam_00720	Ikeda_00028	Karp_02418	Kato_02101	TA686_00758	UT176_00620	UT76-HP_01985
group_6007	46	16S rRNA (adenine(1518)-N(6)/adenine(1519)-N(6))-dimethyltransferase		Boryong_00488	Gilliam_00719	Ikeda_00029	Karp_02419	Kato_02100	TA686_00759	UT176_00619	UT76-HP_01984
group_5824	46	DNA recombination protein RmuC	RmuC	Boryong_00487	Gilliam_00718	Ikeda_00030	Karp_02420	Kato_02099	TA686_00760	UT176_00618	UT76-HP_01983
group_5650	46	zinc metalloprotease		Boryong_00486	Gilliam_00717	Ikeda_00031	Karp_02421	Kato_02098	TA686_00761	UT176_00617	UT76-HP_01982
group_5539	46	outer membrane protein assembly factor BamA	BamA	Boryong_00485	Gilliam_00716	Ikeda_00032	Karp_02422	Kato_02097	TA686_00762	UT176_00616	UT76-HP_01981
group_5649	46	thiol reductase thioredoxin		Boryong_00484	Gilliam_00715	Ikeda_00033	Karp_02423	Kato_02096	TA686_00763	UT176_00615	UT76-HP_01980
group_7769	47	thioredoxin-disulfide reductase	TrxB	Boryong_00020	Gilliam_00024	Ikeda_01757	Karp_00011	Kato_00071	TA686_02375	UT176_00364	UT76-HP_00026
group_7112	47	permease		Boryong_00021	Gilliam_00025	Ikeda_01758	Karp_00012	Kato_00070	TA686_02376	UT176_00363	UT76-HP_00025
group_5621	47	translocation protein TolB	TolB	Boryong_00022	Gilliam_00026	Ikeda_01759	Karp_00013	Kato_00069	TA686_02377	UT176_00362	UT76-HP_00024

group_5775	47	dihydrolipoyl dehydrogenase	lpdA	Boryong_00023	Gilliam_00027	Ikeda_01760	Karp_00014	Kato_00068	TA686_02378	UT176_00361	UT76-HP_00023
group_5425	47	SAM-dependent methyltransferase		Boryong_00024	Gilliam_00028	Ikeda_01761	Karp_00015	Kato_00067	TA686_02379	UT176_00360	UT76-HP_00022
group_5426	47	hypothetical protein		Boryong_00025	Gilliam_00029	Ikeda_01762	Karp_00016	Kato_00066	TA686_02380	UT176_00359	UT76-HP_00021
group_7894	48	type I methionyl aminopeptidase	Map	Boryong_01573	Gilliam_01947	Ikeda_00420	Karp_01288	Kato_01532	TA686_00765	UT176_01751	UT76-HP_01652
group_7905	49	ubiquinone biosynthesis protein UbiB	UbiB	Boryong_01795	Gilliam_02198	Ikeda_00705	Karp_01872	Kato_01873	TA686_00284	UT176_01700	UT76-HP_01485
group_5580	49	ubiquinone biosynthesis protein	UbiJ	Boryong_01796	Gilliam_02197	Ikeda_00704	Karp_01873	Kato_01872	TA686_00283	UT176_01701	UT76-HP_01486
group_6093	49	ribosome maturation factor		Boryong_01797	Gilliam_02196	Ikeda_00703	Karp_01874	Kato_01871	TA686_00282	UT176_01702	UT76-HP_01487
group_6094	49	transcription termination/antitermination protein NusA	NusA	Boryong_01798	Gilliam_02195	Ikeda_00702	Karp_01875	Kato_01870	TA686_00281	UT176_01703	UT76-HP_01488
group_5889	49	translation initiation factor IF-2	InfB	Boryong_01799	Gilliam_02194	Ikeda_00701	Karp_01876	Kato_01869	TA686_00280	UT176_01704	UT76-HP_01489
group_7895	49	ribosome-binding factor A	RbfA	Boryong_01800	Gilliam_02193	Ikeda_00700	Karp_01877	Kato_01868	TA686_00279	UT176_01705	UT76-HP_01490
group_7960	50	preprotein translocase subunit YajC	YajC	Boryong_02000	Gilliam_00087	Ikeda_01330	Karp_00223	Kato_00152	TA686_02313	UT176_00845	UT76-HP_00576
group_6110	50	protein translocase subunit SecD	SecD	Boryong_02001	Gilliam_00088	Ikeda_01331	Karp_00224	Kato_00153	TA686_02312	UT176_00846	UT76-HP_00575
group_8117	51	peptidase S66		Boryong_00304	Gilliam_00580	Ikeda_01592	Karp_00511	Kato_00468	TA686_01761	UT176_00261	UT76-HP_00436

Sample	Genome Length	Length of repetitive sequence (bp)	Percentage of genome which is repetitive
Boryong	2127051	895302	42
FPW1038	2035338	957348	47
Gilliam	2465012	1246424	51
Ikeda	2008987	721214	36
Karp	2469803	1210014	49
Kato	2319449	1050415	45
TA686	2254553	976333	43
TA763	2089396	895735	43
UT176	1932116	635697	33
UT76	2078193	868414	42
REIS	2100092	426115	20
Wilmington	1111496	0	0

Table S5. Total length of repetitive genome sequences in each strain, and as a percentage of the genome. REIS: Rickettsia endosymbiont of Ixodes scapularis. Wilmington: Rickettsia typhi strain Wilmington.

**Product**		**Boryong**	**Gilliam**	**Ikeda**	**Karp**	**Kato**	**TA686**	**UT176**	**UT76**
(p)pGpp hydrolase		37	31	25	40	26	14	16	25
(p)ppGpp synthetase		2	2	1	5	1	0	2	2
spoT ppGpp hydrolase		3	15	7	16	9	11	5	5
ABC transporter ATP-binding protein		1	2	2	2	3	2	1	3
Aconitate hydratase A		1	1	2	1	2	0	1	1
All ankyrin proteins		43	46	40	58	37	39	37	38
	ankyrin	14	26	18	33	23	21	21	25
	Ankyrin repeat-containing protein	13	10	13	11	7	4	10	8
	ankyrin repeat-containing protein 09	4	6	3	4	2	8	3	3
	ankyrin repeat-containing protein 13	3	1	1	1	1	0	1	1
	ankyrin repeat-containing protein 16	9	0	2	7	2	2	1	0
	ankyrin repeat-containing protein 17	0	1	1	1	0	4	1	0
	ankyrin repeat-containing protein 19	0	2	2	1	2	0	0	1
ATP-binding protein		48	85	63	99	91	97	44	87
Cell division protein FtsB		1	2	1	1	1	1	1	1
All conjugal transfer proteins		461	532	378	570	502	462	330	481
	conjugal transfer protein	166	202	138	242	181	202	137	194
	conjugal transfer protein TraA	75	86	60	83	56	64	41	62
	conjugal transfer protein TraC	70	50	39	40	65	37	34	61
	conjugal transfer protein TraD	1	0	2	2	2	2	0	0
	conjugal transfer protein TraG	13	29	21	28	24	25	19	24
	conjugal transfer protein TraH	41	37	37	44	52	34	24	41
	conjugal transfer protein TraI	41	62	32	65	48	50	25	33
	conjugal transfer protein TraN	46	49	30	38	40	27	36	45

	type-F conjugative transfer system pilin assembly protein TrbC	0	0	0	2	0	0	0	0
	type-F conjugative transfer system protein TraW	8	17	19	26	34	21	14	21
deoxyribodipyrimidine photo-lyase		4	1	1	1	1	0	1	0
DNA helicase		0	0	1	3	6	0	0	1
DNA methyltransferase		27	32	17	29	26	22	17	28
DNA polymerase III subunit epsilon		1	1	1	1	1	2	1	1
elongation factor Tu		2	2	2	2	2	2	2	2
exodeoxyribonuclease III		3	1	4	4	2	1	2	3
exodeoxyribonuclease VII small subunit		1	1	1	1	1	2	1	1
Group II intron-encoded protein LtrA		0	0	0	0	0	4	0	0
guanosine polyphosphate pyrophosphohydrolase		3	2	10	3	9	11	1	5
helix-turn-helix domain-containing protein		3	0	0	0	0	4	0	0
histidine kinase		1	8	9	8	13	16	1	9
HNH endonuclease		4	2	1	32	19	37	0	3
hydrolase		5	13	13	11	20	12	7	14
hypothetical protein		321	250	180	259	241	242	134	188
integrase		69	77	69	71	92	87	44	82
All transposases		338	602	306	325	242	409	487	242
	DDE transposase family protein	0	0	4	2	1	3	0	1
	IS110 family transposase	19	8	34	22	14	23	13	5
	IS5 family transposase ISOt6	199	157	101	143	85	163	73	87
	IS630 family transposase	26	342	71	27	37	83	316	29
	transposase	94	95	96	131	105	137	85	120
lipase LipB		1	1	1	1	1	1	0	2
lysine--tRNA ligase		1	1	1	1	1	2	1	1
membrane protein		12	27	17	34	25	20	16	22
N-6 DNA methylase		6	1	0	0	0	0	0	0
NADP-dependent oxidoreductase		1	1	2	1	1	1	1	1
peroxiredoxin		1	2	4	4	7	5	2	2
phosphatidate cytidylyltransferase		1	2	1	1	1	1	1	1
phosphoribosylaminoimidazolesuccinocarboxamide synthase		1	1	1	1	1	3	2	2
polyribonucleotide nucleotidyltransferase		1	1	1	1	1	1	1	2
preprotein translocase SecA subunit-like protein		0	4	2	7	2	9	0	2
Propionyl-CoA carboxylase beta chain		0	1	2	1	2	0	1	1
repeat-containing protein D		4	0	4	1	2	0	1	1
replicative DNA helicase		47	33	28	40	36	39	17	34
reverse transcriptase		58	19	32	5	33	23	2	6
RNA-binding protein		3	2	5	10	3	12	4	4
sodium:proline symporter		4	4	7	6	8	5	5	5
TAL effector protein PthXo1		1	0	3	2	3	0	1	3
All TPR repeat-containing proteins		22	40	18	29	37	24	22	27
	TPR repeat-containing protein 03	0	12	6	8	10	7	11	4
	TPR repeat-containing protein 08	22	28	12	21	27	17	11	23
tryptophan--tRNA ligase		2	1	1	1	1	1	1	1
UDP pyrophosphate synthase		1	2	1	1	1	1	1	1

Table S6. Repeat gene counts in each strain. Repeat genes were grouped by protein similarity and annotated with the product of the longest gene in the group where annotations differed.

Sample	Pseudogenes	Truncated 5'	Truncated 3'	Frameshift
Boryong	432	219	302	46
Gilliam	484	262	278	51
Ikeda	257	141	186	38
Karp	321	105	236	47
Kato	286	143	178	57
TA686	453	200	307	50
UT176	465	107	392	53
UT76	319	149	203	52

Table S7. Pseudogenes and causes of pseudogenisation for each strain. The causes are not mutually exclusive, and may sum to greater than the total number of pseudogenes.

	56kDa	47kDa	MLST	Core genome
56kDa	-	10	10	8
47kDa	10	-	8	6
MLST	10	8	-	10
Core genome	8	6	10	-

Table S8. Robinson-Foulds distances between phylogenetic trees.
